# A need for standardized reporting of introgression: Insights from studies across eukaryotes

**DOI:** 10.1002/evl3.294

**Published:** 2022-07-25

**Authors:** Andrius J. Dagilis, David Peede, Jenn M. Coughlan, Gaston I. Jofre, Emmanuel R. R. D'Agostino, Heidi Mavengere, Alexander D. Tate, Daniel R. Matute

**Affiliations:** ^1^ Biology Department University of North Carolina Chapel Hill NC USA; ^2^ Department of Ecology, Evolution, and Organismal Biology Brown University Providence RI USA; ^3^ Center for Computational Molecular Biology Brown University Providence RI USA; ^4^ Department of Ecology and Evolutionary Biology Yale University New Haven CT USA; ^5^ Department of Ecology and Evolutionary Biology Princeton University Princeton NJ USA

## Abstract

With the rise of affordable next‐generation sequencing technology, introgression—or the exchange of genetic materials between taxa—has become widely perceived to be a ubiquitous phenomenon in nature. Although this claim is supported by several keystone studies, no thorough assessment of the frequency of introgression across eukaryotes in nature has been performed to date. In this manuscript, we aim to address this knowledge gap by examining patterns of introgression across eukaryotes. We collated a single statistic, Patterson's *D*, which can be used as a test for introgression across 123 studies to further assess how taxonomic group, divergence time, and sequencing technology influence reports of introgression. Overall, introgression has mostly been measured in plants and vertebrates, with less attention given to the rest of the Eukaryotes. We find that the most frequently used metrics to detect introgression are difficult to compare across studies and even more so across biological systems due to differences in study effort, reporting standards, and methodology. Nonetheless, our analyses reveal several intriguing patterns, including the observation that differences in sequencing technologies may bias values of Patterson's *D* and that introgression may differ throughout the course of the speciation process. Together, these results suggest the need for a unified approach to quantifying introgression in natural communities and highlight important areas of future research that can be better assessed once this unified approach is met.

## INTRODUCTION

Genome sequencing has revealed that instances of hybridization and introgression—the transfer of genetic materials from one genetic ancestry (i.e., population or species) into a different one—are not rare in nature. Introgression can have myriad effects, and although it is most commonly thought to be deleterious (Martin and Jiggins 2017), introgression may also provide raw genetic materials for adaptation and speciation (Heiser 1973; Rieseberg and Wendel 1993; Dowling and Secor 1997; Arnold and Martin 2009; Suarez‐Gonzalez et al. 2018; Taylor and Larson 2019). Examples ranging from disease vectors (Lee et al. 2013; Fontaine et al. 2015; Norris et al. 2015) to humans (Green et al. 2010) have revealed that allele transfer can be instrumental for range expansion, adaptation, and even speciation. For example, the *EPAS*1 haplotype responsible for Tibetan high‐altitude adaptation is most likely introgressed from Denisovan populations (Huerta‐Sánchez et al. 2014; Racimo et al. 2015). On the other hand, introgressed genes may bear certain costs (Harris and Nielsen 2016)—Neanderthal variants in human populations have been associated with a high health risk for SARS‐CoV‐2 infections (Zeberg and Paabo 2020, 2021). However, the relative importance of introgression for adaptation remains largely unknown, in part because the frequency of introgression across different species also remains unknown.

The susceptibility of genomes to introgression has historically been a subject of lively debate among evolutionary biologists and has persisted well into the genomics age (Heiser 1973; Rieseberg and Wendel 1993; Dowling and Secor 1997; Barton 2001; Mallet 2005; Schwenk et al. 2008; Payseur and Rieseberg 2016). While classically controversial, there is now a general consensus among evolutionary biologists that introgression can occur between taxa. This consensus has in large part been driven by the recognition that species themselves are rarely defined by complete reproductive isolation, so gene flow is possible among a variety of diverging populations, as well as growing genomic evidence of admixture between even highly diverged species (Taylor and Larson 2019; Edelman and Mallet 2021). However, the frequency with which introgression occurs and the genomic and environmental conditions that facilitate or preclude gene exchange between species are unresolved. Nonetheless, there are good reasons to believe introgression may vary in frequency across the tree of life as well as over the course of speciation, as illustrated by examining the conditions that must be met for introgression to occur. We discuss each of these in turn.

For introgression to take place, hybrids must first form and then be able to serve as a bridge for genetic material to cross species boundaries. Thus, introgression requires at least a degree of sympatry and incomplete prezygotic isolation. As a result, taxa with larger range overlap or weaker mate choice are expected to show higher rates of hybridization and potentially higher rates of introgression. Furthermore, the hybrids must be viable at least to the age of reproduction and be partially fertile to produce advanced backcrosses. While hybrid fitness is expected to decrease as species continue to diverge (Prager and Wilson 1975; Coyne and Orr 1989; Coughlan and Matute 2020; Satokangas et al. 2020), it is possible that introgression occurs rather freely until a critical threshold of low fitness in hybrids is developed (Barton 2001; Roux et al. 2016). Since the rate at which reproductive isolation evolves differs widely by taxa (Coughlan and Matute 2020), there may likewise be differences in the degree of introgression between species. Several landmark reviews have examined the frequency of hybridization in general (Knobloch 1972; Dowling and Secor 1997; Payseur and Rieseberg 2016), but none to our knowledge have examined introgression specifically.

While individual studies across focal taxa have been instrumental in revealing specific instances of introgression, the relative occurrence of introgression across taxa remains unknown. To address the differences in introgression across taxa, a comparative approach that consolidates measurements of introgression is needed. The probability of ongoing migration has been elegantly analyzed for some taxa by Roux et al. (2016), but no systematic analysis of introgression has been performed across multiple kingdoms of eukaryotes. The difficulty, in part, has been in quantifying introgression—while shared haplotypes or reduced divergence within a particular region are evidence for potential introgression between two species, they are difficult to compare between species. As researchers moved from sequencing individual genes to entire genomes, novel methods to quantify the degree of introgression have been developed. One of the earliest and most successful is Patterson's *D* (Green et al. 2010; Durand et al. 2011), the first of what have collectively been called *f‐*statistics (Reich et al. 2009). These statistics evaluate the degree to which allele frequencies or tree topology patterns support introgression versus incomplete lineage sorting by looking for asymmetry in the frequency of derived allele sharing between sets of species (Figure S1). In the case of Patterson's *D*, this is measured as the difference in derived alleles shared between taxa P1 and P3 and P2 and P3, where P1 and P2 are sister. Patterson's *D* therefore has immediate blind spots – as it requires a population/species pair in which only one branch has experienced introgression, it is unable to detect introgression between two sister species, and because it relies on an asymmetry in the number of shared derived alleles, introgression from P3 into both P1 and P2 will lead to nonsignificant or small values. The significance of Patterson's *D* is also hard to evaluate, with both jackknife and bootstrap approaches having drawbacks, as no null expectation for Patterson's *D* given the demography and population structure of each lineage exists. Finally, Patterson's *D* is also known to be affected by both the timing and direction of introgression (Martin et al. 2015), so it has spawned a series of other statistics (see Table S1). These different statistics and approaches have been compared in other literature, and Patterson's *D* in general is one of the poorer estimators for the fraction of the genome that has introgressed (Martin et al. 2015; Hibbins and Hahn 2018; Hahn and Hibbins 2019; Hamlin et al. 2020). Nonetheless, due to both its simplicity and ease of calculation, Patterson's *D* has become an extremely common test for introgression.

While care must be applied when evaluating any of the *f‐*statistics, they represent an opportunity to compare the frequency and strength of evidence for introgression across different taxa. Ideally, *f‐*statistics would be computed for a variety of taxa using a single set of approaches, as has been done by Hamlin et al. (2020) and Singhal et al. (2021), but it is difficult to scale this approach using comparable data across eukaryotic life. Alternatively, published data can be used to investigate differences in introgression across taxa. In this manuscript, we undertake the latter approach.

By searching through 724 studies published since 2005 with claims of introgression, we extracted 33,178 *f*‐statistics from 123 studies. The vast majority of the records we obtained were of Patterson's *D*. While Patterson's *D* is not a precise estimator of the fraction of the genome that has introgressed, it is at least proportional to this quantity, and so pairs of species in which large fractions of the genome show evidence for introgression should show larger Patterson's *D* values than those where only small portions of the genome have introgressed (Martin et al. 2015; Pfeifer and Kapan 2019; Hamlin et al. 2020). The resulting dataset was used to ask whether there were differences in introgression between taxa and how evidence for introgression is impacted by sequencing technology, genetic divergence, and several life‐history traits. While we identify several intriguing patterns, our meta‐analysis exposes the need for clearer reporting criteria for introgression studies, as well as further efforts at comparative work in introgression.

## METHODS

### Search criteria

To create a comprehensive list of papers from which we could extract Patterson's *D* values, we performed a Web Of Science search. We first searched for papers that contained the terms “introgression,” “hybrid,” and “genomic” and complemented the results with any papers citing any of the two papers that defined major *f*‐statistics (Green et al. 2010; Martin et al. 2015). Due to the relative breadth of our initial search criteria, we captured many papers on experimental introgression lines, hybrids occurring solely in the lab, methods to detect introgression or hybridization, and many perspectives and reviews. Papers were then manually inspected for claims of introgression, resulting in nearly 724 papers with claims of introgression. These papers were annotated for the major taxonomic group of the study organism as well as the types of evidence provided when introgression was confirmed. The list of these contributions appears in Supplementary File 1.

### Extracting *f*‐statistics and criteria for inclusion

We next examined any papers with at least one of the *f*‐statistics (Table S1) to extract data. We excluded any papers in which *f‐*statistics were only presented for specific genomic windows, rather than genome‐wide, as well as studies that only presented *f*‐statistics in figures (Figure S2). For each study, we extracted the populations under study, their reported *f*‐statistic and its value, and reported significance. Due to a high variability in which statistics were reported, we also annotated the genomic data type (whole genome sequencing (WGS), reduced representation sequencing (RRS), i.e., RAD or GBS, transcriptome/exome, amplicon sequencing) used for the study as well as whether the authors reported all possible *f*‐statistics, only significant ones, or a specific subset of interest, as well as whether multiple outgroups were used.

The resulting dataset was largely composed of values of Patterson's *D*, with a small number of $\hat{f_{d}}$ and *F*_4_ values and a handful of observations of other *f*‐statistics (Figure S2). As a result, we use Patterson's *D* for all downstream analyses. Patterson's *D* ranges between −1 and 1, with significant negative values indicating introgression between P1 and P3, while significant positive values indicate introgression between P2 and P3 (Figure S1). Since the majority of the data were arranged in a fashion such that Patterson's *D* values were positive, we standardized this across the dataset, swapping the identities of P1 and P2 when Patterson's *D* was negative, and using the absolute value of Patterson's *D*. Since some studies report Patterson's *D* between all possible triplets of populations, we next annotated the data to identify tree topologies that represent the most conservative estimate of introgression (or “nontreeness” (Malinsky et al. 2018)). For each unique set of three taxa, we labeled the topology such that ((P1,P2),P3) reported the smallest value of Patterson's *D*. The filtering of the data is outlined in Figure S2.

We next annotated our records using a custom pipeline available on GitHub (https://github.com/adagilis/introgression_meta) along with the compiled dataset. For each record, the NCBI taxonomic id of the relevant species/populations was identified using the *rentrez* package in R 4.0.3 (Team 2020), followed by manual spot checking and correction. We used these IDs to further annotate the data for phylogenetic classification (kingdom, phylum, class, and family of each introgression event) and to download sequence data to calculate genetic distances between species pairs. We calculated genetic distances in several different ways. In line with traditional approaches, we first downloaded all sequences of a single gene (either *COI* or *ITS1* and *ITS2* or *CYTB*) from ncbi's nucleotide database (Sayers et al. 2022) for each species. For each species pair, we aligned the sequences using the G‐INC algorithm in *mafft* version 7.407 (Katoh and Standley 2013) and calculated Jukes‐Cantor distances between the species using the *dna.dist* function of the R package *ape* (Paradis and Schliep 2019; Team 2020). This resulted in 15,865 records with annotated genetic distances between P2 and P3, with many introgressed species pairs missing sequences for either of the genes of interest. Additionally, introgression between species at these particular genes would lead to vast underestimations of genetic distances between them. We therefore used a second approach to estimate genetic distance. We downloaded up to 10,000 sequences from the NCBI nucleotide database for each species in the pair. Reciprocal best BLAST hits (Camacho et al. 2009) from the two species’ sequences were then aligned using mafft, and average Jukes‐Cantor distance was calculated for the resulting alignments in R 4.0.3 using the ape package. This method uses different genes to measure genetic distance between different species pairs, but we were able to annotate a total of 26,351 records with genetic distance using this approach. The two measures are broadly correlated (Figure S3) across all phyla except plants. We repeated all model fits with either reciprocal best hit or single gene distances, with single gene distance model results reported in supplementary figures.

Using these genetic distances, we next labeled records that likely broke the assumptions of Patterson's *D*. For each record, we asked if the genetic distance between P2 and P3 was smaller than that between P2 and the putative outgroup, P4. If this was the case, it was likely that an inappropriate outgroup was selected, and these records were excluded from further analysis. We did not perform this step for intraspecific introgression, as the same species was often used for all of P1, P2, P3, and P4.

Finally, we identified records with significant *f*‐statistics. As a variety of reporting criteria were used, we did this in several ways. In studies that claimed to only report significant *f*‐statistics, all records were labeled significant. In studies that reported *P*‐values, records with *P *< 0.05 were labeled significant. Finally, in studies that only reported *Z* scores, records with an absolute *Z* score value above 2 were labeled significant. We did not account for multiple comparisons within each study, since some studies only reported species pairs with a significant signal of introgression, while others reported all pairwise comparisons; however, we attempted to account for differences in reporting between studies with our mixed model fitting.

### Model fitting and phylogenetic correction

We fit a series of models to test for differences in introgression between taxa, test for the effects of genetic distance between introgressed species pairs, and examine the effects of sequencing technology as well as outgroup choice. To account for the random effects stemming from differences in reporting and power of different genomic sequencing, we include the source study for each value as a random effect. In this approach, the random effect of study accounts for nearly half of the residual variation in observed Patterson's *D* values (Tables S2–S18). However, as each study was generally limited to an individual taxon, this conservative approach is likely to underpower our ability to detect meaningful differences between biological groups—any real biological differences are captured as an effect of reference. All models used the *lme4* package in *R* (Bates et al. 2015; Team 2020), while pairwise comparisons between fixed effects were performed using the *emmeans* package (Lenth 2020).

Correcting for phylogenetic effects in introgression studies can be done in several ways. Since *f*‐statistics are a property of a set of tips, rather than a particular branch, traditional approaches such as phylogenetically independent contrasts are not possible. Mixed model approaches that include genetic distance as a random effect may work, but our dataset spans eukaryotes, and calculating a genetic distance matrix for the entire dataset was not feasible. A more straightforward mixed modeling approach is to include species identity as a random effect and genetic distance between species as a fixed effect. We do this for several models described in the following section. However, we were also interested in the effects of genetic distance on introgression in general. For this case, we cannot use genetic distance to both account for phylogenetic effects and to measure its direct effect on introgression. To address this issue, we performed two sets of analyses. First, we fit a mixed model, resampling the data such that at most one instance of each species was included, generating phylogenetically bootstrapped model fits. This method is in principle quite similar to using species identity as a random effect but may be overly conservative (note also that the random effect of species pairs is dropped in the bootstrap fits). Problematically, since introgression can occur in the ancestor of many descendant species with measures of Patterson's *D*, even including each species at most once does not fully account for phylogenetic nonindependence. We therefore also reduced our data to a parsimonious set of independent introgression events. We clustered all introgression observations into phylogenetically independent sets of taxa, such that for each set of taxa, there was at least one significant report of introgression between each species in the set and at least one other. We then calculated the average genetic distance between all species with reports of introgression in the set and averaged Patterson's *D* for the set. This approach resulted in 100 phylogenetically independent clusters of introgression.

#### Effect of taxonomic group on introgression

Models 1–3 test for a relationship between taxonomic groups and the significance of reports of Patterson's *D*. Model 1 assumes no effect of genetic distance, while models 2 and 3 incorporate a fixed effect term for either reciprocal best hit distance or single gene distance (*COI*, *ITS* or *CYTB*), respectively. Models 4–9 test the relationship between taxonomic groups and the magnitude of Patterson's *D* (all models) and incorporate reciprocal best hit distance (models 5 and 8) or single gene distance (models 6 and 9). Models 7–9 limit the data to observations from classes with at least 2 studies to mitigate for the potential effect of individual studies.

#### Effects of genetic distance between introgressed pairs and distance to outgroup

Models 10–15 examine the effects of genetic distance on either the significance (models 10 and 11) or magnitude (models 12–15) of Patterson's *D*. We first asked if the distance between P2 and P3 to the outgroup played a significant role in either the significance or magnitude of Patterson's *D* (models 10–13) and then examined whether the distance between the introgressed species pairs was related to the magnitude of Patterson's *D* using either metric of genetic distance (models 14 and 15).

#### Effect of sequencing type

Models 16 and 17 examined the effect of sequencing type (reduced representation, transcriptome/exome, or whole genome sequence) on the magnitude of Patterson's *D*. Genetic distance as a fixed effect and species pair identity as a random effect were included to account for phylogenetic nonindependence. The sequencing type was annotated based on the data type reported in the paper.

## RESULTS

**Figure 1:**
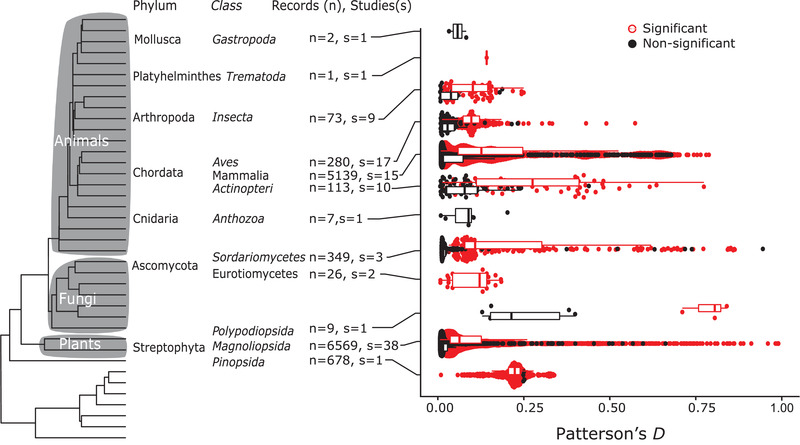
Distribution of Patterson's *D* values sampled across the Eukaryote phylogeny of phyla (Hedges et al. 2015), with only phyla with labeled data. The number of records per taxonomic class (n) and number of source studies (s) are listed by the taxon labels, while the distribution of Patterson's *D* values is displayed on the inset. Significant values of Patterson's *D* (determined either by *P*‐value, *Z* score, or significance stated in source paper) are colored black, while nonsignificant values are colored red.

Through our literature search, we identified 724 papers with claims of introgression since 2015 (Supporting Information S1). From these papers, 130 used some form of *f*‐statistics, and we were able to extract *f*‐statistics from 121 of the studies. This resulted in 33,464 records of *f*‐statistics, 32,191 of which were of Patterson's *D*. These statistics were further filtered based on criteria set out in the methods (and see Figure S2) for a total of 13,250 records from 99 studies that were used to fit various models, 9564 of which were statistically significant in their original studies. The distribution of these values is shown in Figure 1.

### Differences in introgression by kingdom, phylum, and class

One of the oldest debates in speciation genetics is whether plants and animals differ in their propensity to produce hybrids (Rieseberg and Wendel 1993; Chen et al. 2018). This argument can be extended to a more inclusive taxonomic base: does the amount of introgression differ across taxonomic groups? To address this question, we fit a series of mixed models to determine whether different taxonomic groups showed differences in reported Patterson's *D*. We first asked if different groups are more or less likely to report significant introgression in the form of significant Patterson's *D*. We then asked if the magnitude of Patterson's *D* reported among significant introgression events differs between taxa. We focused on the kingdom, phylum, and class categories for fixed effects on reported Patterson's *D*. In total, we fit 13 linear models to study the differences in introgression between taxa. We performed post hoc ANOVA to test for significant factors and used least‐squares means to obtain pairwise differences in marginal means between different taxa. Table 1 lists the models, and Tables S2–S18 show the results for these linear models, significance of individual terms using Satterthwaite's method implemented in the *lmerTest* package and pairwise comparisons between groups using the least‐squares means approach implemented in the *emmeans* package.

**Table 1:** Summary of Models. Each model was subsequently bootstrapped using our custom phylogenetic bootstrap approach such that each species appeared at most once as either P2 or P3 in the subsampled data

Model	Outcome	Fixed Effects	Random Effects
Model 1	Significance (Pat *D*)	kingdom/phylum/class (k/p/c)	reference + species pair
Model 2	“ ”	k/p/c + genetic distance (rbh)	“ ”
Model 3	“ ”	k/p/c + genetic distance (coi/its/cytb)	“ ”
Model 4	Magnitude of Pat *D*	k/p/c	“ ”
Model 5	“ “	k/p/c + genetic distance (rbh)	“ ”
Model 6	“ “	k/p/c + genetic distance (coi/its/cytb)	“ ”
Model 7	“ “ (only classes with >1 study)	k/p/c	“ ”
Model 8	“ “ (only classes with >1 study)	k/p/c + genetic distance (rbh)	“ “
Model 9	“ “ (only classes with >1 study)	k/p/c + genetic distance (coi/its/cytb)	“ ”
Model 10	Significance	gen. distance (rbh) * gen. distance to outgroup (rbh)	“ ”
Model 11	Significance	gen. distance (coi/its/cytb) * gen. distance to outgroup (coi/its/cytb)	“ ”
Model 12	Magnitude	gen. distance (rbh) * gen. distance to outgroup (rbh)	“ ”
Model 13	Magnitude	gen. distance (coi/its/cytb) * gen. distance to outgroup (coi/its/cytb)	“ ”
Model 14	Magnitude	gen. distance (rbh)	“ ”
Model 15	Magnitude	gen. distance (coi/its/cytb)	“ ”
Model 16	Magnitude	sequencing type + gen. distance(rbh)	“ ”
Model 17	Magnitude	sequencing type + gen. distance(coi/its/rbh)	“ “

Genetic distance, genetic distance between P2 and P3; rbh, calculated using reciprocal best hits between species; coi/its/cytb, calculated using single genes (either *ITS*, *COI*, or *CYTB*); species pair, unique code for each combination of P2 and P3.

Across models that only include nested effects for kingdom, phylum, and class, models supported some significant differences between groups in magnitude, but not significance, of reported Patterson's *D* (Figures S5, S8, and S11). On the kingdom level, studies of plants report significantly larger values of Patterson's *D* than either studies of animals or fungi (Figures S8 and S11, and Tables S5 and S8). Class‐level differences are driven by Polypodiopsida (ferns) and Pinopsida (conifers)—two plant classes with Patterson's *D* values from only a single study each. While insignificant in our model fits, differences between Actinopteri (ray‐finned fishes minus bichirs) and the other classes within vertebrates have large effect sizes even when performing phylogenetic bootstraps (Figures S8–S13), and it is worth noting that significance is not straightforward to evaluate in mixed models. Including genetic distance modified the significance of individual groups (Figures S8 vs S9, and S10 and S11 vs S12 and S13), but effect sizes remained largely consistent even with the inclusion of genetic distance across bootstraps. Furthermore, plant records had the fewest genetic distances annotated with many taxa lacking these sequences on the NCBI nucleotide database, so lack of significance when including genetic distance may be driven by the reduction in observations. To further minimize the potential effects of individual studies, we also fit the models excluding data from taxonomic classes with fewer than 2 studies each. We found statistically significant differences between plants and animals when not including genetic distance (and marginally significant when using reciprocal best hits), suggesting that previously observed effects were not driven entirely by ferns and conifers (Tables S8, S9, and S10). Effect sizes are large and deviate from 0 in phylogenetic bootstraps (Figures S11–S13) for many of the same groups as in prior models, suggesting that some differences may also be robust to study/reporting biases.

### Genetic distance between species pairs impacts evidence for introgression

One of the expectations of hybridization is that as divergence increases between the parental species, the number of incompatibilities increases at a fast pace (Orr 1995; Turelli and Orr 2000; Satokangas et al. 2020). Since the probability of hybridization and the fraction of the genome that can introgress has been hypothesized to be affected by the density of hybrid incompatibilities (Veller et al. 2019), hybridization and subsequent backcrossing between more divergent species should lead to lower signals of introgression (Wiens et al. 2006; Hamlin et al. 2020). We tested this in two ways. First, we examined how the probability of an *f*‐statistic being significant changes as the distance between species pairs increases. However, as it is likely that many pairs of species with no significant introgression will go unreported, we also tested whether the magnitude of Patterson's *D* among species pairs with significant evidence of introgression changes with increasing divergence between species. We annotated our dataset with genetic distances between pairs of species using two different approaches—either using *COI*, *ITS*, or *CYTB* sequences to calculate Jukes Cantor genetic distances or using reciprocal best BLAST hits between species to calculate Jukes Cantor. We then fit mixed models including the genetic distance between species with evidence for introgression (P2 to P3) as well as the average distance between these species and the outgroup used (P2 and P3 to P4) as fixed effects.

**Figure 2:**
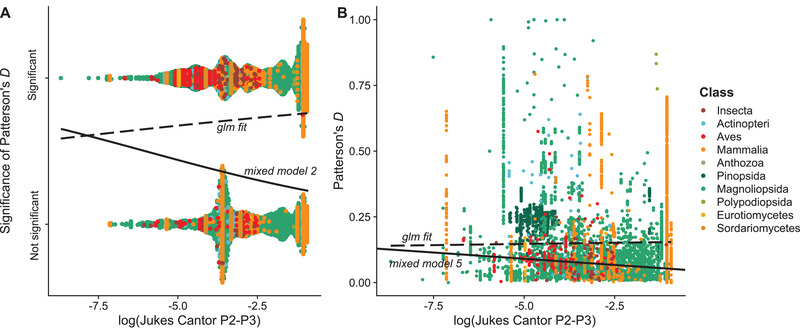
The relationship between genetic distance and A) Significance of introgression tests and B) Magnitude of Patterson's D. Both relationships are significant in linear mixed models, but phylogenetic bootstrap estimates of effects overlap 0 for significance of Patterson's *D* (Figure S6) while remaining significant for the magnitude of Patterson's *D* (Figure S9). Solid lines represent the best fit from mixed models, while dashed lines show naïve linear model fits – accounting for the random effects of species pairs and reference reverses the slope in both cases.

**Figure 3:**
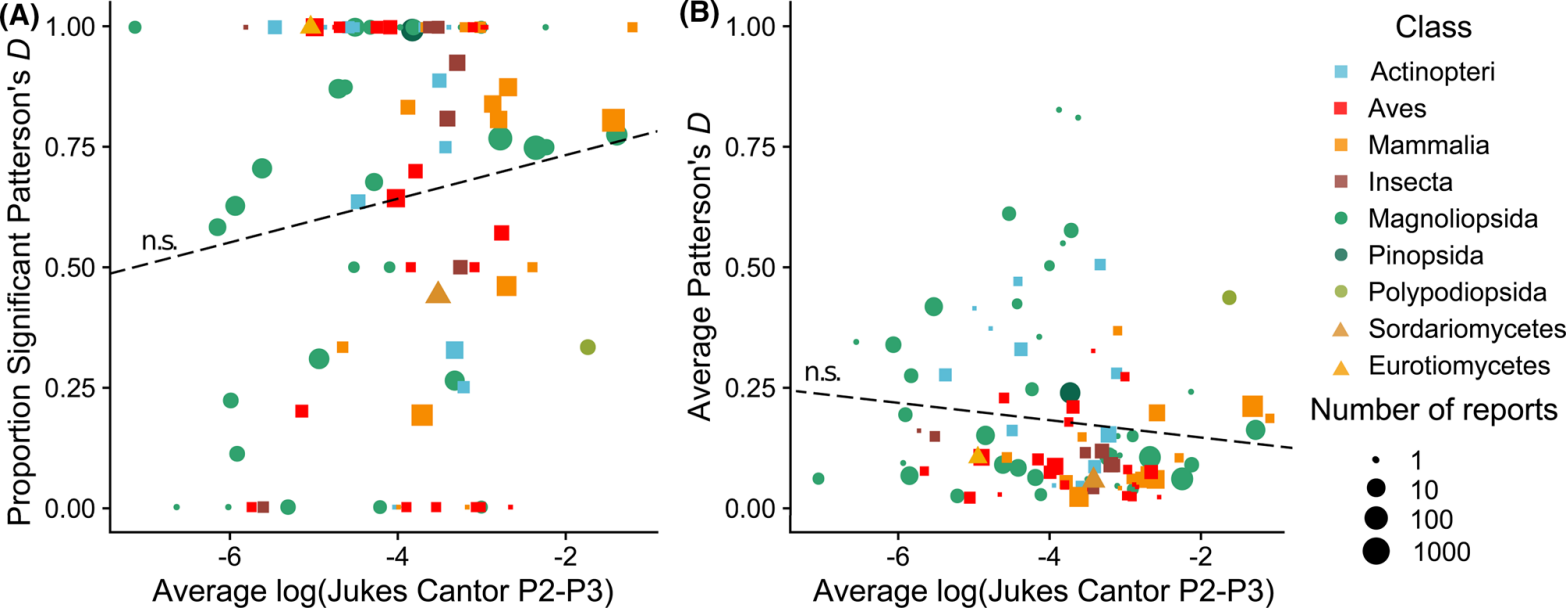
Phylogenetically independent clusters of introgression reports versus genetic distance. Reducing the data to 100 clusters of introgression events, we find no significant relationship between genetic distance and either significance or magnitude of Patterson's *D*. Dashed lines show linear model fits (inclusion of reference as a random effect was precluded by some clusters consisting of reports from many references).

First, we found that distance to the outgroup is a significant predictor of significance, but not of magnitude of Patterson's *D* (Figures S14–S17), with more distant outgroups leading to more likely significant introgression events. Second, we find statistically significant effects of genetic distance on both the significance and magnitude of Patterson's *D* (Figure 2). Phylogenetic bootstrapping does not support these effects on the significance of Patterson's *D*, but they do for the magnitude of reported Patterson's *D* in some models (Figures S18–S19). While promising, our bootstrapping approach may be prone to issues due to ancient introgression being reported as multiple different introgression events between many species’ pairs. We therefore averaged genetic distances and Patterson's *D* across 100 phylogenetically independent clusters of introgression, and we found no significant relationship between genetic distance and either the significance or magnitude of Patterson's *D* (Figure 3). Despite these results, in all models tested, the inclusion of genetic distance was overwhelmingly likely to be the most significant effect (Tables S2–S18) and again suggests either a potential relationship or strong phylogenetic signal for introgression.

Next, we explored whether there was heterogeneity in the relationship between the magnitude of Patterson's *D* and the genetic distance between the hybridizing species across more granular taxonomic groups. We fit a mixed model with an interaction between taxonomic order and Jukes Cantor on the observed Patterson's *D* value, with random effects of each study and species pair. The results, summarized in Figure S4, demonstrate that order‐specific slopes are supported for only a handful of taxa (significant taxon‐specific slopes shown with filled‐in labels), with increasingly weaker (smaller slope) relationships as more data are available per order. A similar approach for larger taxonomic units (classes and phyla) does not support phylum‐ or class‐specific slopes (data not shown). While taxonomic differences in introgression patterns are difficult to disentangle from noise due to low sampling and other systemic biases, we also note that within each taxonomic class, orders with more samples tend to show more negative slopes. More sampling is needed to further elucidate differences between taxa.

### Data type and Patterson's *D*

One of the potential sources of error in detecting introgression is bias due to the type of sequencing employed to detect it. Reduced representation sequencing (RRS), such as RAD, ddRAD, or GBS, subsamples a smaller proportion of the genome than methods such as transcriptome/exome sequencing, which again represent a smaller subset of the genome than whole genome sequencing (WGS). For each study included in our data, we classified the source data as coming from RRS, transcriptome/exome or WGS and fit a mixed model with genome data type and genetic distance as fixed effects and source study as random effects. Even when bootstrapped to include each species at most once (Figure S19 and Table S17), this model identifies significant effects of genetic data type on reported Patterson's *D*. Specifically, a significant difference is supported between records coming from studies using RRS and those using WGS. Transcriptome/exome records were intermediate to both of the other groups and did not differ significantly from either. Using single gene distances, these effects are not observed (Figure S20 and Table 18), but very few records from RAD studies were annotated using the single gene approach.

## DISCUSSION

One of the most enduring debates in evolutionary biology has been whether speciation can proceed with gene flow. More recent forms of debate have taken the form of asserting that introgression might be a common feature of evolution (Seehausen 2004; Mallet et al. 2016). To generally answer this question requires compiling data and timing of gene flow between species across a variety of taxa. While we cannot directly answer the question of the prevalence of gene flow in speciation, our meta‐analysis demonstrates that introgression has left a mark on the genomes of extant populations and is supported by a vast number of studies across multiple eukaryotic systems. We find that there is extensive variation in the amount of introgression across taxa, but we are unable to distinguish between real biological differences and differences in study effort, reporting, or computational approaches. We discuss each of these considerations as follows.

### Variation across taxa

Our data suggest several broad taxonomic patterns. Among significant Patterson's *D* values, plant studies report significantly larger Patterson's *D* than either fungi or animals. Within plants, this pattern seems to be most strongly driven by studies of ferns and conifers (classes Polypodiopsida and Pinopsida), each represented by a single study, but some effects remain even when only classes with more than a single study are used. Effect sizes among our models also indicate differences between fish (class Actinopteri) and many other taxa. The latter observation is consistent with previous work suggesting that fish have the highest rates of hybridization of any vertebrate taxa and examples of hybridization between very old taxa (Schwenk et al. 2008; Rothfels et al. 2015), both suggesting at least more potential opportunities for introgression among fish. While further study is necessary, these differences could be driven by interesting differences in biology. There are good reasons why taxa with faster rates of developing reproductive isolation, for instance, mammals, compared to birds and anurans (Wilson et al. 1974; Prager and Wilson 1975; Fitzpatrick 2004; Coughlan and Matute 2020; Matute and Cooper 2021), may show less evidence for introgression. Faster speciation leads to a smaller time frame in which successful hybridization between divergent subspecies can occur. More rapid speciation also means that there are fewer fixed differences in introgress between species and more maintained ancestral polymorphism, increasing the ratio of incomplete lineage sorting (ILS) to introgression in statistics such as Patterson's *D*. On the other hand, rapid speciation may also be associated with increased introgression either due to introgression driving speciation or rapid radiations leading to weak postzygotic barriers in the resulting species complex (Mallet et al. 2016). Speciation rates vary heavily both between phylogenetic classes and orders and within them (Rabosky 2009; Rabosky et al. 2013; Schluter and Pennell 2017; Coughlan and Matute 2020). Furthermore, there is a great degree of variation in the amount of sympatry (Nosil 2013; Matute and Cooper 2021) and overall hybridization rates (Chen et al. 2018; Mitchell et al. 2019). Finally, the definition of species also varies across eukaryotes (Coyne and Orr 2004; Matute and Sepúlveda 2019), so the difference may be in part due to differences in how different fields label species and not speciation rates per se. Thus, we expect to see a variation in Patterson's *D* across eukaryotes, at varying scales, due to a variety of phenomena.

By linking observed Patterson's *D* values with genetic distances calculated from publicly available data, we were able to ask several questions about how patterns of introgression scale with divergence. First, we find a negative relationship between genetic distance and both the significance and magnitude of Patterson's *D* (Figure 2). While this result is largely expected from several theoretical perspectives (Hamlin et al. 2020; Singhal et al. 2021), this relationship is weak overall in our study. As species diverge, the build‐up of reproductive isolation presents both fewer opportunities for introgression (Jagoda et al. 2018; Petr et al. 2019) and increases the selection against introgressed regions (Staubach et al. 2012; Jagoda et al. 2018; Petr et al. 2019). However, for several reasons further explained in the caveats, the relationship may disappear due to bias in reporting, choice of study systems, and a lack of distinction between ongoing and ancient introgression. In line with these caveats, the relationship between genetic distance and significance of Patterson's *D* overlaps 0 when we bootstrap by sampling each species from the data at most once to control for phylogenetic nonindependence and a high degree of pseudoreplication. The relationship with genetic distance is more complex, with phylogenetic bootstraps overlapping 0, when distance to outgroup is included as an effect (Figures S16 and S17) but remains significant after bootstrapping when it is the only fixed effect included (Figures S18 and S19). However, this overall weak pattern may also be driven by looking for a single slope of introgression vs genetic distance across all studied taxa, with different slopes canceling each other out. This intuition is supported by significant relationships of genetic distance when allowing for individual intercepts for each taxon (Models 2, 3, 5, 6, 8, and 9).

To further examine the possibility of taxon‐specific relationships between introgression and genetic distance, we calculated the slope of the best‐fit linear models between genetic distance and Patterson's *D* across individual taxonomic orders (Figure S4). Unsurprisingly, the number of observations in an individual taxon played a strong role in determining the slope of the relationship between genetic distance and Patterson's *D*. As more data become available for any order, the slope becomes less steep, but many orders show positive, rather than negative, relationships between Patterson's *D* and genetic distance. Primates, for example, have a fairly strong signal for increased divergence leading to increased evidence for introgression, but this is likely biased by the heavy focus on ancient introgression in hominids, giving many positive values of Patterson's *D* for relatively highly diverged species pairs. Two major lines of inquiry are suggested by these data. First, the effects of ancient introgression in determining the relationship of Patterson's *D* and genetic distance need to be explored. While the general expectation has been for an overall decrease in introgression as taxa diverge (Roux et al. 2016; Hamlin et al. 2020), it is possible that this signal is swamped by ancient introgression or that for some taxa introgression is more likely between diverged species pairs. Second, it seems that there may be genuine differences in the relationship between genetic distance and introgression among some of the best studied taxa (Figure S4), a pattern that, to our knowledge, has not been previously reported or expected. These differences may be driven by study effort differences but also due to the evolutionary history of introgression or differences in the process of speciation between taxa.

### Recent versus ancient introgression

Our dataset is unable to distinguish between ongoing/recent and ancient introgression. Even when Patterson's *D* is applied correctly, it captures both ancient and recent introgression events. Ancient introgression in a taxon may lead to many species pairs with positive Patterson's *D* values reported in our dataset (see Pines (*Pinopsida*), for instance), while a recent introgression event is likely to be represented by just a single species pair. This generates a potential bias for elevated Patterson's *D* between more diverged populations, as Patterson's *D* identifies signals of introgression between pairs of species rather than at a particular branch/timepoint. By clustering together introgression events in the same set of species, we can somewhat minimize this effect, but our estimates of the timing of introgression are still inaccurate. The solution for the comparative biology of introgression is to identify not only the proportion of introgression but also the timing. Several methods are making progress on this front (Edelman et al. 2019; Martin and Amos 2021; Svedberg et al. 2021), while some *f*‐statistics approaches can identify likely introgression timing given a tree topology (Malinsky et al. 2018). The future of the field thus may be better able to deal with some of the caveats we discuss next.

### Caveats and record limitations

**Figure 4:**
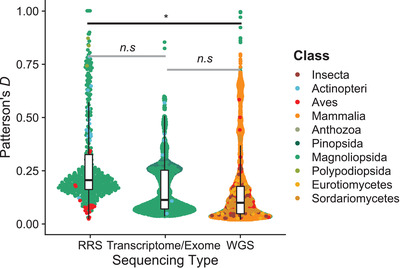
Sequencing type and Patterson's *D*. Studies using reduced representation sequencing (GBS/RAD) report significantly larger values of Patterson's *D* than studies that use whole genome sequences.

Our results are not devoid of caveats. One of the main findings of our analyses is the extensive variation in the depth and quality of reports claiming support for introgression. While the field has uniformly moved toward the study of introgression using genome sequences, not all studies have used whole genome analyses. This is due to the extreme genome size of some taxa (Gregory 2021) and because there are potential trade‐offs in the number of individuals sequenced and the amount of genome sequenced. The benefits and caveats of reduced sequencing have been described elsewhere (Puritz et al. 2014; Lowry et al. 2017), but briefly, the selection of markers invariably biases estimates of introgression, as reduced representation sequencing (RRS) data inherently underestimate true levels of diversity (Gautier et al. 2013; Cariou et al. 2016). In our data, we detected a significant difference in the magnitude of reported Patterson's *D* based on the sequencing technology used (Figure 4), but these effects are hard to disentangle from potential reporting differences or real biological differences between taxa. It is possible that simply due to the smaller number of sites, larger differences in ABBA and BABA sites are necessary for statistically significant results in reduced representation approaches. Since publication favors reporting of statistically significant results, this could lead to inflated Patterson's *D* values among studies using approaches with fewer reported sites.

A second noteworthy caveat pertains to the limitations of Patterson's *D* itself. First, because of its very proposition, Patterson's *D* detects excess introgression into one species. If the donor species contributes the same alleles to two sister species, then Patterson's *D* will be zero. The metric may be inflated when either of the P1 or P2 taxa has experienced a bottleneck, it produces false positives under certain demographic scenarios, and it is exceptionally hard to distinguish introgression from the P3 taxon compared to an unsampled “ghost” taxon (Martin et al. 2015; Hibbins and Hahn 2021; Tricou et al. 2022). It is important to note that ghost introgression represents true introgression; however, it misidentifies the source and thus may be misleading in comparative studies. Because the evolutionary remnants of introgression are likely to occur in blocks along the genome (generating blocks of high/low values of Patterson's *D*), but such blocks can also be generated by demographic processes, evaluating its statistical significance is also not straightforward—not all statistically significant values of Patterson's *D* will likely represent true introgression events. Finally, the statistic relies on a specific species tree being true—when the test is applied to populations that may not meet the topology expectation, it is likely to return meaningless values. Since species relationships in taxa with a high degree of introgression are hard to determine (root node of Neoaves, for example, Prum et al. 2015), such errors might ironically be more prevalent for taxa in which introgression actually has occurred and may be hopelessly difficult in cases of introgression‐driven speciation. Among our data, we frequently filter observations that either use a topology that reports a larger Patterson's *D* than some other topology (39 studies) and/or topologies that may be incorrect based on our genetic distance metrics (47 studies) (Figure S2). While we exclude these records in our data analysis, the true topology for the species must be known to measure a meaningful Patterson's *D* or many of the other *f*‐statistics.

These caveats extend our analysis of the relationship between genetic distance and Patterson's *D*. First, Patterson's *D* does not accurately measure the proportion of introgression, which is expected to decrease with increasing genetic distance. It is a test for the presence of introgression and is proportional to the fraction of the genome that has introgressed under some circumstances, but whether it should decline over genetic distances is unclear. Second, our dataset consists of a variety of introgression events, some recent or ongoing, others quite ancient. Since we measure genetic distance between contemporary samples, we may therefore be overestimating the distance between the species pairs when introgression occurred. Our data are also depleted for small/zero values of introgression, both because researchers are unlikely to measure introgression between distantly related taxa that are not expected to have a history of hybridization and because researchers are unlikely to report small or insignificant values of Patterson's *D* due to the “file‐drawer effect” (Scargle 1999). A systematic analysis of the file‐drawer effect was impeded by differences in statistical significance reporting between studies (with some studies reporting only Z scores for individual records, for instance), but note that of the 32,116 Patterson's *D* values we extracted, only 6,653 (20%) were nonsignificant. The file‐drawer effect may also explain the lack of a strong relationship between Patterson's *D* and genetic distance – researchers are unlikely to report small values of Patterson's *D* between highly diverged species, as introgression between them is not expected, and similarly may not measure introgression between very closely related species/populations because gene flow in those cases is less interesting.

Perhaps the largest difficulty does not pertain to Patterson's *D* itself but to how the results of the tests are reported. The lack of consistency in reporting introgression paints a muddled picture of its frequency and any differences between taxa. In terms of reporting, a variety of approaches are used—in some cases, researchers report only those values of introgression statistics that represent the particular set of introgression events under study. In others, only significant values of statistics are reported (which naturally leads to a depletion of low values of Patterson's *D*). Sometimes a mix of approaches is used, where only some particular sets of species are tested for introgression and only some values are reported. We argue that reporting all possible Patterson's *D* values given the groups under study would facilitate future comparative studies. We strongly suspect that the banding observed in Figure 4 and others is caused by the subjective application of significance thresholds. This issue is related to the potential of a “file‐drawer effect,” with differing values across papers being considered significant enough to report. Last, it is nearly impossible to disentangle differences in reporting and study effort between fields from actual differences in introgression frequency between systems. While studies such as this one are helpful to identify general trends, until the field unites behind a unified reporting standard (see “A unified reporting standard” below), truly comparative studies that use a single set of approaches to interrogate introgression across taxa will be necessary.

### Directions for the field

Alongside the development of our understanding that speciation is a process and not an event has come to the appreciation of the potential for ongoing gene flow between what are often believed to be good species. Introgression, rather than an exceptional occurrence, seems to be a common feature of evolution in eukaryotes, at least in cases where it has been examined. Studies across a wide array of eukaryotes are now shedding light on the frequency of introgression. However, several developments are necessary to understand the drivers of introgression.

#### A unified reporting standard

To answer the overarching question of “How prevalent is introgression across the tree of life?,” researchers could shift their focus from taxa‐centric studies of introgression to more clade‐centric studies (e.g., Hamlin et al. (2020), Malinsky et al. (2018), Edelman et al. (2019), Suvorov et al. (2021), Small et al. (2020), Singhal et al. (2021)). However, we recognize that this may not always be possible or feasible. Alternatively, we suggest the following unified reporting standard to further advance the field's abilities to perform comparative analyses of introgression across the tree of life. We first suggest avoiding the use of Patterson's *D* or similar statistics when the species tree is highly uncertain, as these statistics are contingent on a known topology. For all pairwise comparisons that do not violate the assumed species tree, we recommend that researchers report a genome‐wide Patterson's *D* value, number of ABBA, BABA, and BBAA sites. Although Patterson's *D* has its shortcomings, it is very simple to compute (see ANGSD (Korneliussen et al. 2014), scikit‐allel (Miles 2020), or D‐suite (Malinsky et al. 2021)) and can be calculated from population genetic data as well as whole genome alignment data, which makes it applicable to test for the presence of introgression on both population level and phylogenetic time scales. Additionally, we recommend that researchers assess significance using either a standard block jackknife procedure—as first described in Reich et al. (2009)—or a bootstrap approach and subsequently report both the standard error and corresponding Z score. Second, we suggest that researchers calculate a genome‐wide *D_P_* value (Hamlin et al. 2020) for all possible pairwise comparisons of groups that do not violate the assumed species tree topology. *D_P_* is simple to calculate given the number of ABBA, BABA, and BBAA sites and has been shown to be a much more accurate predictor of the proportion of the genome that has introgressed. We would also like to emphasize that this unified reporting standard should not replace any new methods to detect and/or quantify introgression but instead provide the minimum and necessary information to empower future comparative studies. Indeed, new methods to quantify the timing of introgression are likely to increase the power of comparative studies by identifying the timing and direction of introgression.

## CONCLUSIONS

Our goal with this piece is not to become the last word on the question of the prevalence of introgression across taxa in nature. Instead, we provide a state‐of‐the‐art compilation that reveals the current understanding of the field, tests current hypotheses, and, most importantly, highlights the most significant gaps in the field. We find that introgression has been identified across eukaryotes, but sampling is uneven, and reporting needs to be standardized to allow for comparative questions in introgression to be answered. Although our dataset is not able to answer these questions, we find several patterns that motivate further study.

## AUTHOR CONTRIBUTIONS

A.J.D., J.M.C., and D.R.M. conceived the study. All authors participated in data gathering and annotation. A.J.D. and D.P. performed the data analysis. A.J.D., D.R.M., J.M.C., and D.P. wrote the initial draft of the manuscript. All authors edited the final version of the manuscript.

Associate Editor: Z. Gompert

## Supporting information

File S1. List of papers examined for these data.
Table S1. List of introgression summary statistics that were collected
Tables S1‐S18: Attached as a separate file. Model fit outputs for model 2–17.
Figure S1 Patterson's *D* expected under various scenarios.
Figure S2 Breakdown of extracted *f*‐statistics used in this study.
Figure S3 Correlation between genetic distances measured either through reciprocal best blast hit sequences, or alignments of single focal genes (*COI*, *ITS* or *CYTB*, average taken when more than one available).
Figure S4 Smaller slopes among orders with more data.
Figure S5 Mixed model effect sizes for Model 1, only effects of taxonomy included.
Figure S6 Mixed model effect sizes for Model 2.
Figure S7 Mixed model effect sizes for Model 3.
Figure S8 Mixed model effect sizes for Model 4.
Figure S9 Mixed model effect sizes for Model 5.
Figure S10 Mixed model effect sizes for Model 6.
Figure S11 Mixed model effect sizes for Model 7.
Figure S12 Mixed model effect sizes for Model 8.
Figure S13 Mixed model effect sizes for Model 9.
Figure S14 Mixed model effect sizes for Model 10.
Figure S15 Mixed model effect sizes for Model 11.
Figure S16 Mixed model effect sizes for Model 12.
Figure S17 Mixed model effect sizes for Model 13.
Figure S18 Mixed model effect sizes for Model 14.
Figure S19 Mixed model effect sizes for Model 15.
Figure S20 Mixed model effect sizes for Model 16.
Figure S21 Mixed model effect sizes for Model 17.

Supplementary Tables S2‐118

## Data Availability

All scripts/data used for analyses and to generate plots are available on request and will be made available on Dryad prior to publication. Versions of the scripts and data are also available at https://github.com/adagilis/introgression_meta.
