# Supplementary material for: A need for standardized reporting of introgression: Insights from studies across eukaryotes
Source: Evol Lett. 2022 Jul 25;6(5):344–57. doi: 10.1002/evl3.294 (PMC9554761; doi:10.1002/evl3.294)
Supplement: Supplementary file 1 — File S1. List of papers examined for these data. Table S1. List of introgression summary statistics that were collected Tables S1‐S18: Attached as a separate file. Model fit outputs for model 2–17. Figure S1 Patterson's D expected under various scenarios. Figure S2 Breakdown of extracted f‐statistics used in this study. Figure S3 Correlation between genetic distances measured either through reciprocal best blast hit sequences, or alignments of single focal genes (COI, ITS or CYTB, average taken when more than one available). Figure S4 Smaller slopes among orders with more data. Figure S5 Mixed model effect sizes for Model 1, only effects of taxonomy included. Figure S6 Mixed model effect sizes for Model 2. Figure S7 Mixed model effect sizes for Model 3. Figure S8 Mixed model effect sizes for Model 4. Figure S9 Mixed model effect sizes for Model 5. Figure S10 Mixed model effect sizes for Model 6. Figure S11 Mixed model effect sizes for Model 7. Figure S12 Mixed model effect sizes for Model 8. Figure S13 Mixed model effect sizes for Model 9. Figure S14 Mixed model effect sizes for Model 10. Figure S15 Mixed model effect sizes for Model 11. Figure S16 Mixed model effect sizes for Model 12. Figure S17 Mixed model effect sizes for Model 13. Figure S18 Mixed model effect sizes for Model 14. Figure S19 Mixed model effect sizes for Model 15. Figure S20 Mixed model effect sizes for Model 16. Figure S21 Mixed model effect sizes for Model 17. [file EVL3-6-344-s002.pdf]

**Table 1: Summary of Models.** Each model was subsequently bootstrapped using our custom phylogenetic bootstrap approach such that each species appeared at most once as either P2 or P3 in the subsampled data. Genetic distance = genetic distance between P2 and P3, rbh= calculated using reciprocal best hits between species, coi/its/cytb = calculated using single genes (either *ITS*, *COI* or *CYTB*), species pair = unique code for each combination of P2 and P3.

| Model | Outcome | Fixed Effects | Random Effects |
|---|---|---|---|
| Model 1 | Significance (Pat *D*) | kingdom/phylum/class (k/p/c) | reference + species pair |
| Model 2 | “ ” | k/p/c + genetic distance (rbh) | “ ” |
| Model 3 | “ ” | k/p/c + genetic distance (coi/its/cytb) | “ ” |
| Model 4 | Magnitude of Pat *D* | k/p/c | “ ” |
| Model 5 | “ “ | k/p/c + genetic distance (rbh) | “ ” |
| Model 6 | “ “ | k/p/c + genetic distance (coi/its/cytb) | “ ” |
| Model 7 | “ “ (only classes with >1 study) | k/p/c | “ ” |
| Model 8 | “ “ (only classes with >1 study) | k/p/c + genetic distance (rbh) | “ “ |
| Model 9 | “ “ (only classes with >1 study) | k/p/c + genetic distance (coi/its/cytb) | “ ” |
| Model 10 | Significance | gen. distance (rbh) * gen. distance to outgroup (rbh) | “ ” |
| Model 11 | Significance | gen. distance (coi/its/cytb) * gen. distance to outgroup (coi/its/cytb) | “ ” |
| Model 12 | Magnitude | gen. distance (rbh) * gen. distance to outgroup (rbh) | “ ” |
| Model 13 | Magnitude | gen. distance (coi/its/cytb) * gen. distance to outgroup (coi/its/cytb) | “ ” |
| Model 14 | Magnitude | gen. distance (rbh) | “ ” |
| Model 15 | Magnitude | gen. distance (coi/its/cytb) | “ ” |
| Model 16 | Magnitude | sequencing type + gen. distance(rbh) | “ ” |
| Model 17 | Magnitude | sequencing type + gen. distance(coi/its/rbh) | “ “ |

**SUPPLEMENTARY MATERIAL**

**File S1. List of papers examined for this data. Papers matching our search criteria are listed, with annotations of claims of introgression, data used to support the claims, and**

For Review Only

**whether the paper was excluded from data extraction if it did have claims of introgression, as well as the reason for exclusion.**

**Table S1. List of introgression summary statistics that were collected**

| Statistic | Infers | Requirements | Citation |
|---|---|---|---|
| $Patterson's\ D$ | The presence of introgression. | Four whole genome sequences from (((P1, P2), P3), Outgroup) population tree. | (Green et al. 2010; Durand et al. 2011) |
| $D_{FOIL}$ | The presence and direction of introgression. | Five whole genome sequences from ((P1, P2), (P3, P4), Outgroup) population tree. | (Pease and Hahn 2015) |
| $D_3$ | The presence of introgression. | Three whole genome sequences from ((P1, P2), P3) population tree. | (Hahn and Hibbins 2019) |
| $D_{FS}$ | The presence, direction, timing, and rate of introgression. | Two whole genome sequences for the P3 and outgroup taxa and sufficient sampling of whole genome sequences for the P1 and P2 taxa from the (((P1, P2), P3), Outgroup) population tree. | (Martin and Amos 2021) |
| $\hat{f}$ | The admixture proportion. | Four whole genome sequences from (((P1, P2), P3), Outgroup) population tree. | (Durand et al. 2011) |
| $\widehat{f_d}$ | The admixture proportion. | Sufficient sampling of whole genome sequences for the P2 and P3 taxa from the (((P1, P2), P3), Outgroup) population tree. | (Martin et al. 2015) |
| $\widehat{f_{dM}}$ | The admixture proportion. | Sufficient sampling of whole genomes sequences for the P1, P2, and P3 taxa from the (((P1, P2), P3), Outgroup) population tree. | (Malinsky et al. 2015) |
| $\widehat{f_{hom}}$ | The admixture proportion. | Four whole genome sequences from (((P1, P2), P3), Outgroup) population tree. | (Martin et al. 2015) |
| $d_f$ | The admixture proportion. | Four whole genome sequences from (((P1, P2), P3), Outgroup) population tree. | (Pfeifer and Kapan 2019) |
| $D_p$ | The admixture proportion. | Four whole genome sequences from (((P1, P2), P3), Outgroup) population tree. | (Hamlin et al. 2020) |

**Tables S2-S20:** Attached as a separate file. Model fit outputs for model 2-19.

For Review Only

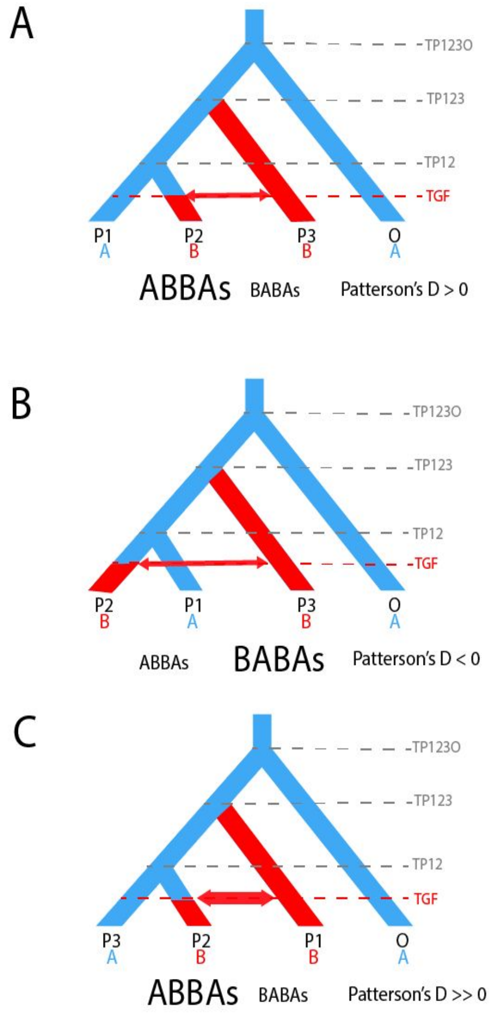


**Figure S1: Patterson's *D* expected under various scenarios**. In all panels lineages in blue carry the ancestral allele and are represented by an A, lineages in red carry the derived allele and are represented by a B, TP123O represents the time when the outgroup population diverged from the ancestral population P123, TP123 represents the time when P3 diverged from the ancestral population P12, TP12 represents the time when P1 and P2 diverged, and TGF represents the time when gene flow occurred. A) The true species relationship is shown, with sister taxa P1 and P2, and introgression between P3 and P2. Introgression increases the shared derived alleles between the two populations (ABBAs), leading to an overall positive Patterson's D. B) If P2 and P1 are swapped, Patterson's D becomes negative, so changing the sign of negative Patterson's D and swapping the identity of P1 and P2 allows for easier comparisons. C) When the wrong tree topology is used, Patterson's D values can be highly misleading. In this case, P1 and P2, which are actually sister taxa, will share many derived alleles, leading to a highly elevated Patterson's *D*. When multiple arrangements of a triplet are available, we

assume the arrangement with the lowest Patterson's *D* is most likely to be the "true" species tree.

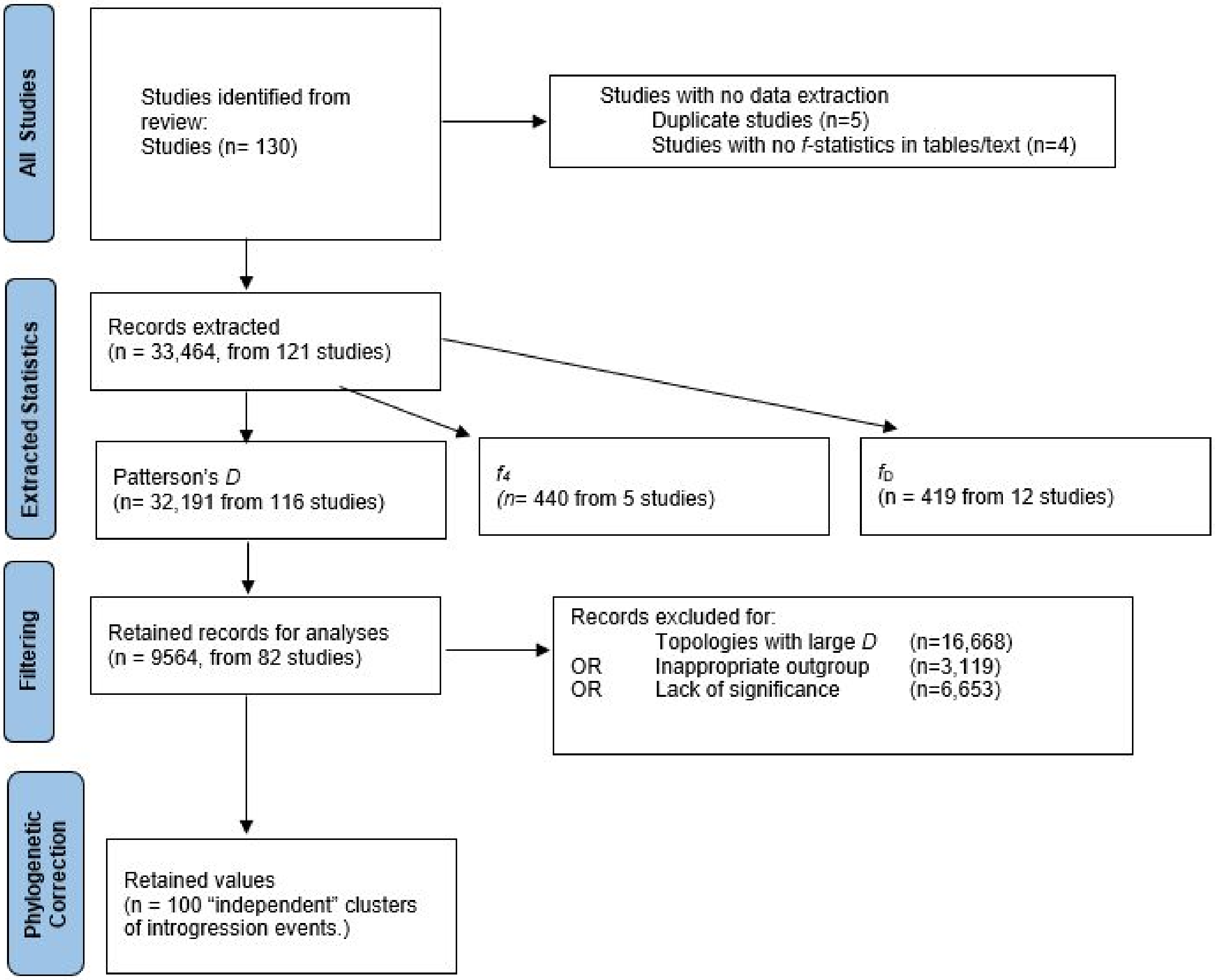


**Figure S2: Breakdown of extracted *f*-statistics used in this study. A total of 130 studies with *f*-statistics were examined for data extraction. Of these, 121 studies contained some *f*-statistics, and 3 main *f*-statistics were extracted. The vast majority of observations were of Patterson's *D*, which were used for all analyses. These were then filtered for cases where 1) the topology of P1, P2, P3 minimized Patterson's *D*, 2) An outgroup was used as P4 and 3) observations were significant. Finally, the retained values were subset to a phylogenetically independent set of Patterson's D observations.**

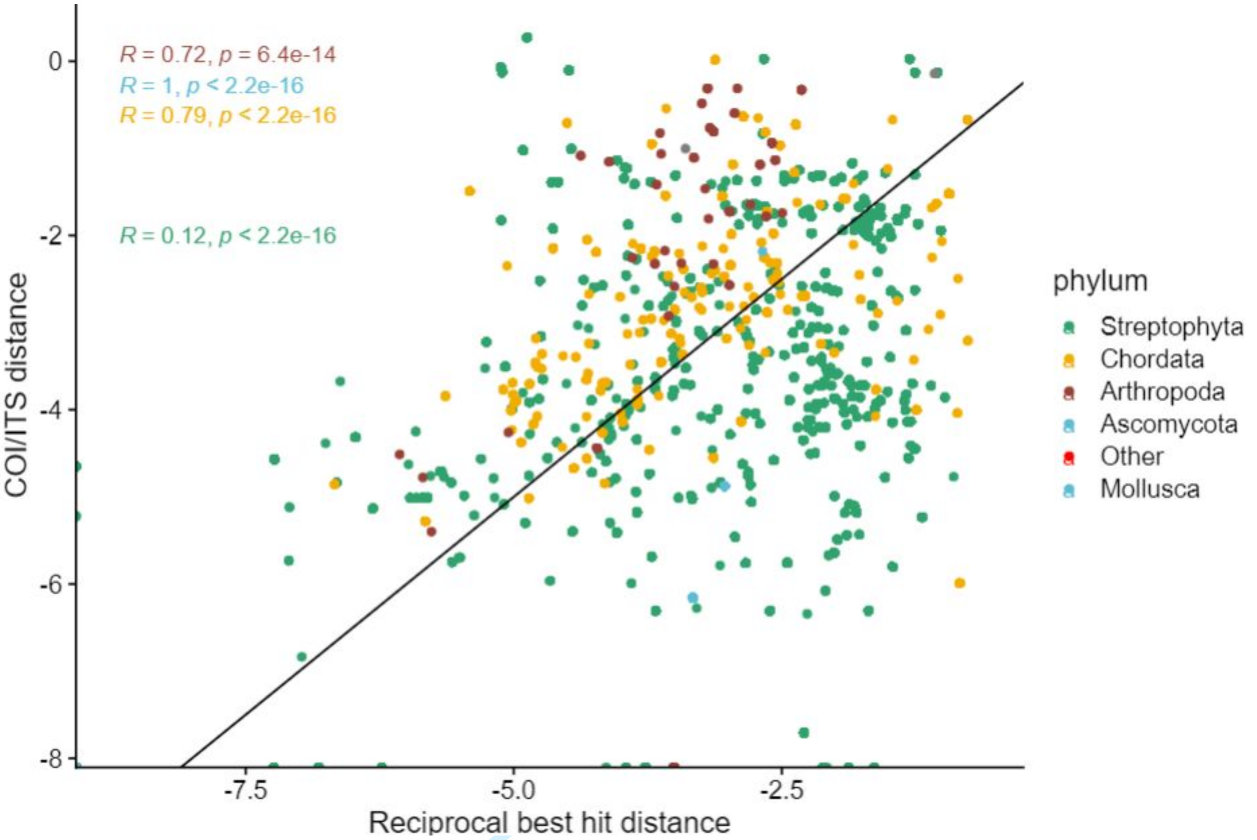


**Figure S3: Correlation between genetic distances measured either through reciprocal best blast hit sequences, or alignments of single focal genes (*COI*, *ITS* or *CYTB*, average taken when more than one available).**

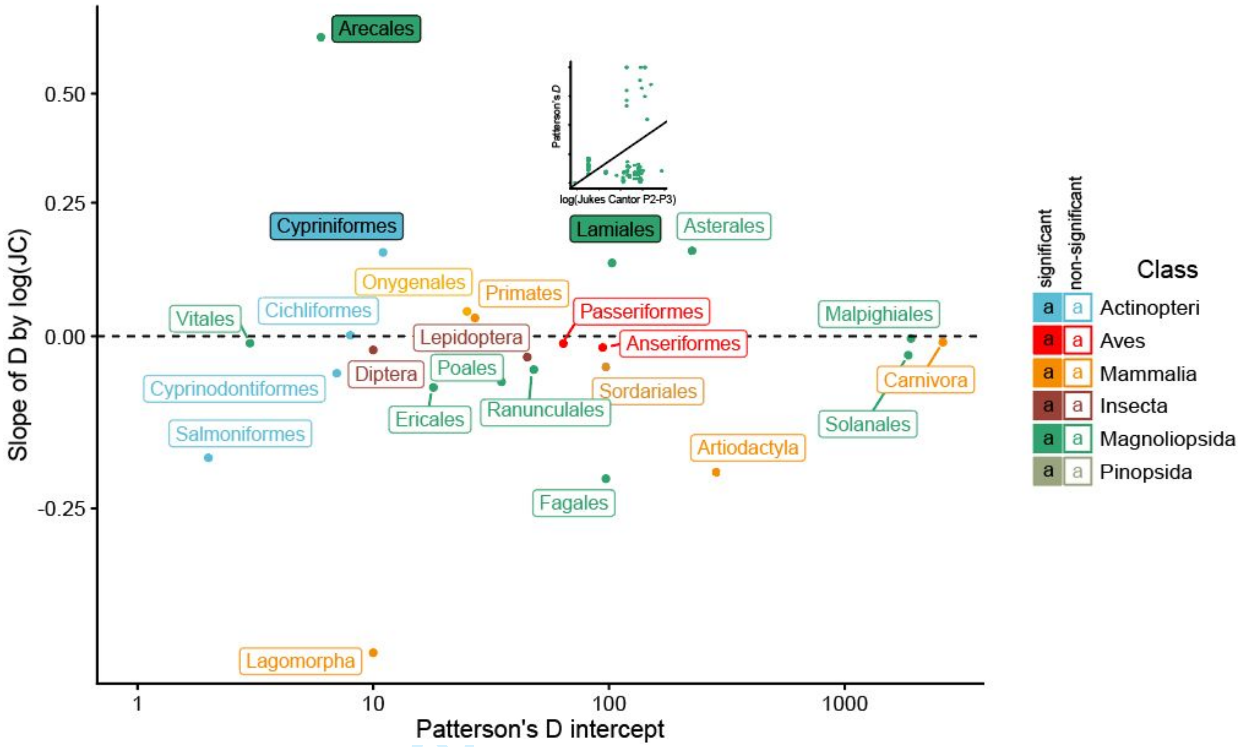


**Figure S4: Smaller slopes among orders with more data. A mixed model with an interaction between genetic distance and taxonomic order was fit, and the results are summarized as the taxon specific slope vs the number of records for the order. Broadly, orders with more comparisons have weaker relationships between Patterson's *D* and genetic distance, but only three orders support an order specific relationship. Inset shows relationship for one of the significant groups- Lamiales.**

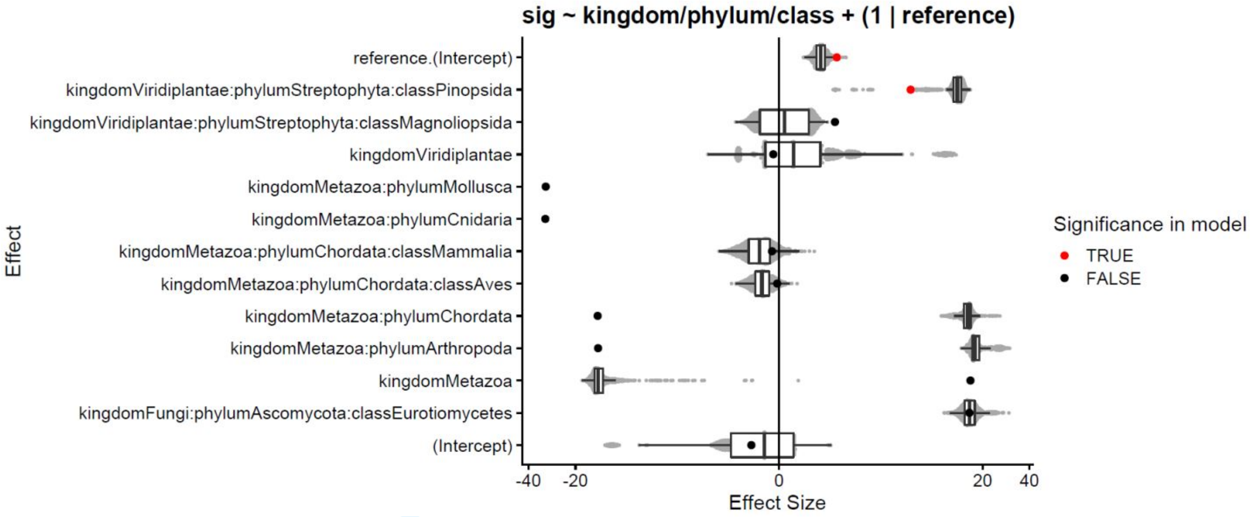


**Figure S5: Mixed model effect sizes for Model 1, only effects of taxonomy included. Significance in model, evaluated using the lmerTest package, is indicated by color of points (black = non significant, red = significant). Grey points and box plots indicate bootstrap fits and ranges, respectively. Bootstraps were performed by re-sampling the data so that each species appeared at most once as either P2 or P3. Some effect sizes do not overlap with 0 in bootstraps, but are not significant in the model either due to small sample sizes for the groups or random effects of reference. In this model, Animal Patterson's *D* reports are significantly less likely to be significant, while among animals Chordata and Arthropoda report significant Patterson's *D* much more frequently than Cnidarians.**

or Review Only

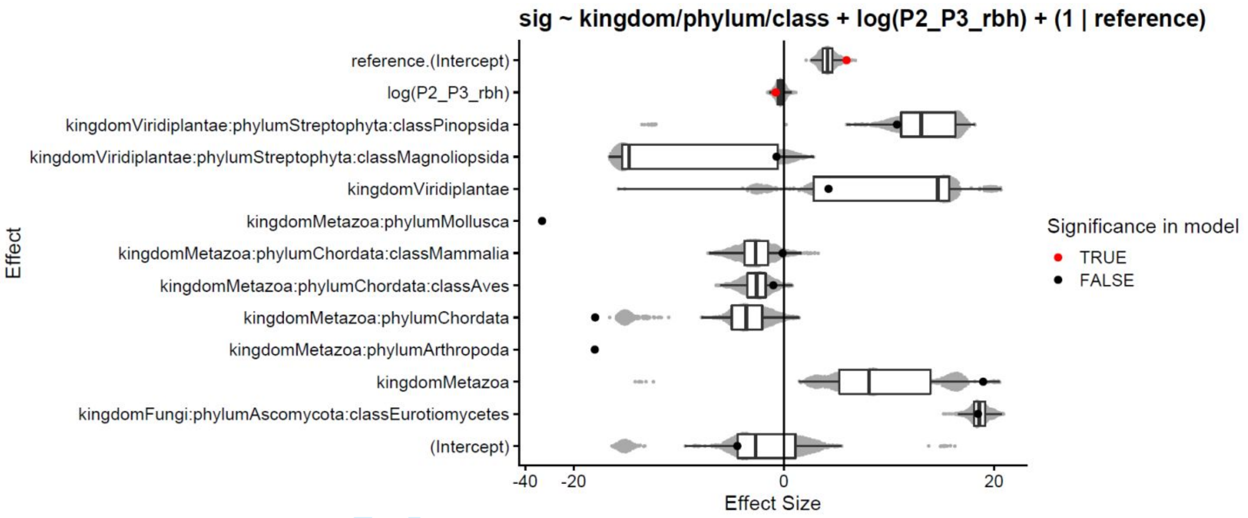


**Figure S6: Mixed model effect sizes for Model 2. In this model, Jukes Cantor between P2 and P3 has a significant negative effect on likelihood of a report being significant, however, phylogenetic bootstrap effect estimates overlap with no effect. Labels as in Figure S5.**

For Review Only

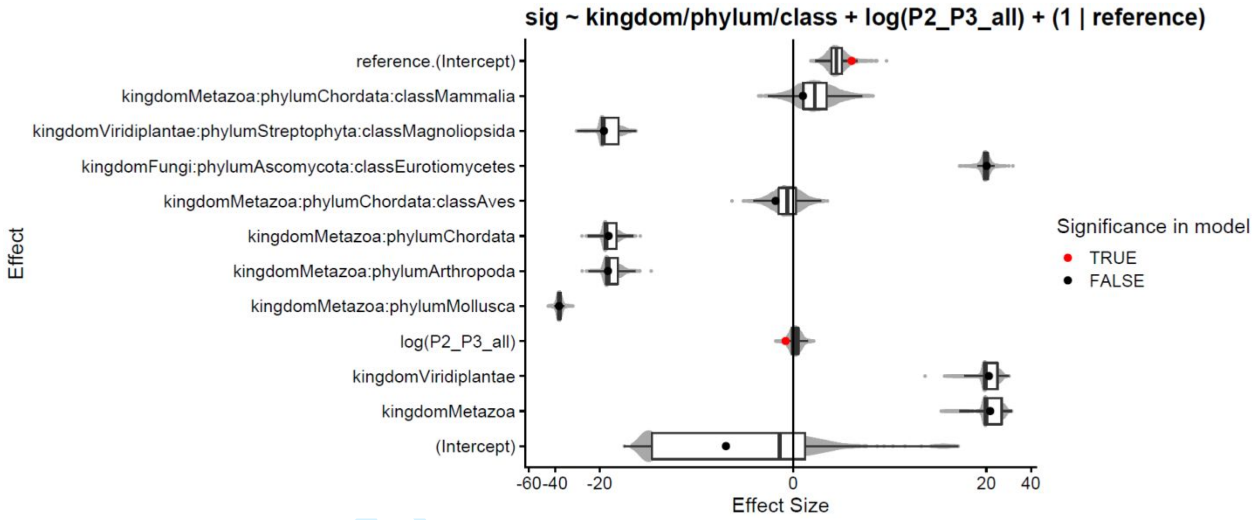


**Figure S7: Mixed model effect sizes for Model 3. Using *COI*, *ITS* or *CYTB* to measure Jukes Cantor, we see results analogous to Model2. Labels as in Figure S5.**

For Review Only

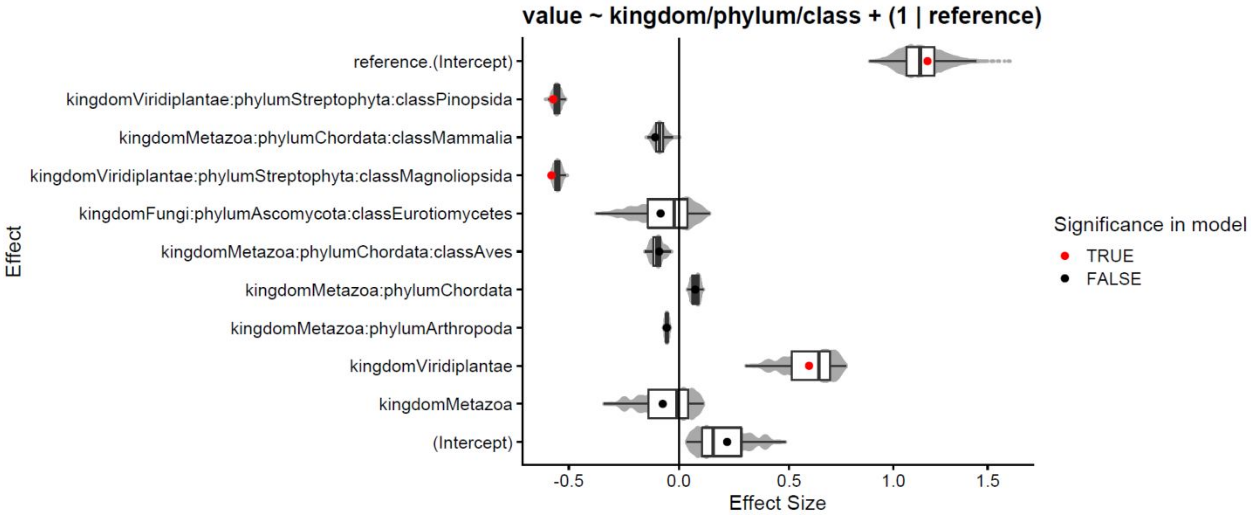


**Figure S8: Mixed model effect sizes for Model 4. Plant (Viridiplantae) reports have significantly higher Patterson's D than either Fungi or Animals (Metazoa). This is driven in part by very large reported Patterson's *D* in Ferns (Polypodiopsida), significantly larger than Magnoliopsida. While non-significant, Pinopsida also shows negative effect sizes for Patterson's D vs. Polypodiopsida, and both Mammals and Birds (Aves) show lower Patterson's D than Fish (Actinopteri). Labels as in Figure S5.**

For Review Only

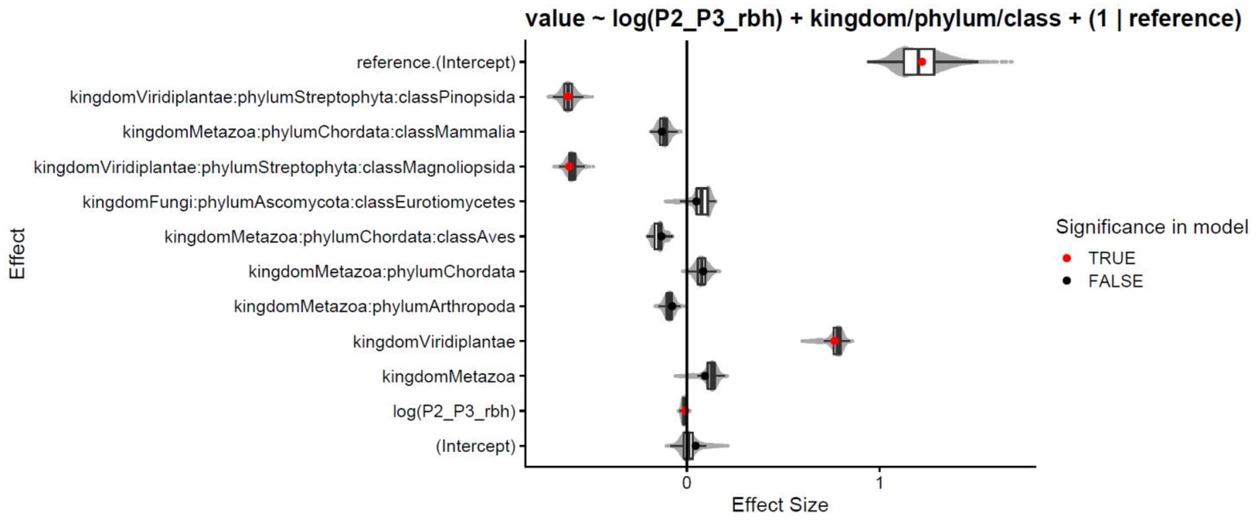


**Figure S9: Mixed model effect sizes for Model 5. Including genetic distance does not change the results of Model 4, with the same significant comparisons as before, but also a significant negative relationship between genetic distance and Patterson's *D*, even after bootstrapping. Labels as in Figure S5.**

For Review Only

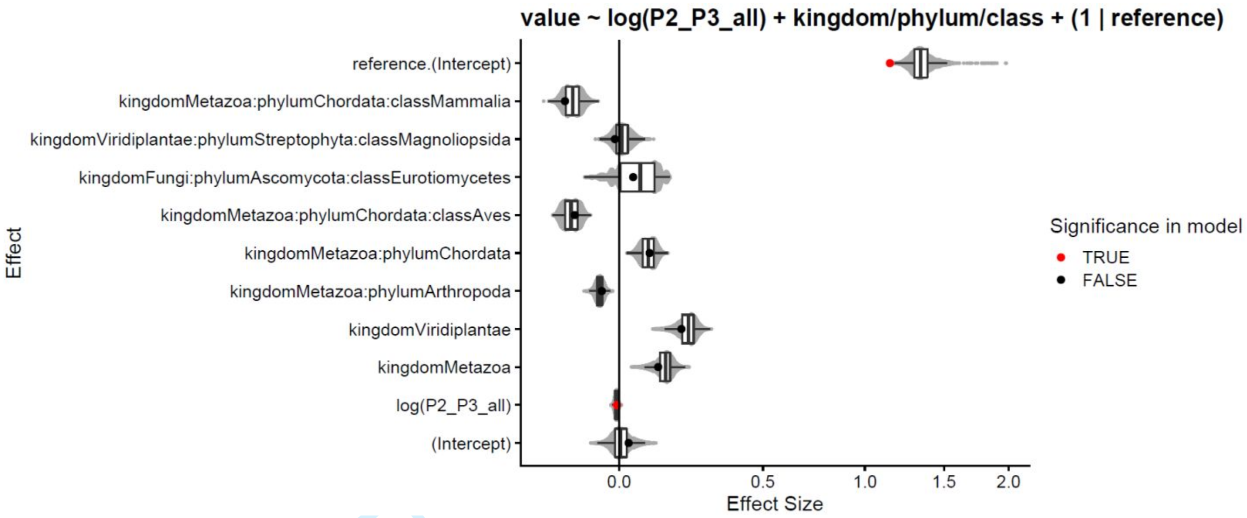


**Figure S10: Mixed model effect sizes for Model 6. Using *COI*, *ITS* or *CYTB* genetic distances rather than reciprocal best hits removes significance of taxonomic effects, in part due to lack of these genetic distances for many taxa. Note, however, that effect sizes are similar to the previous model. Labels as in Figure S5.**

For Review Only

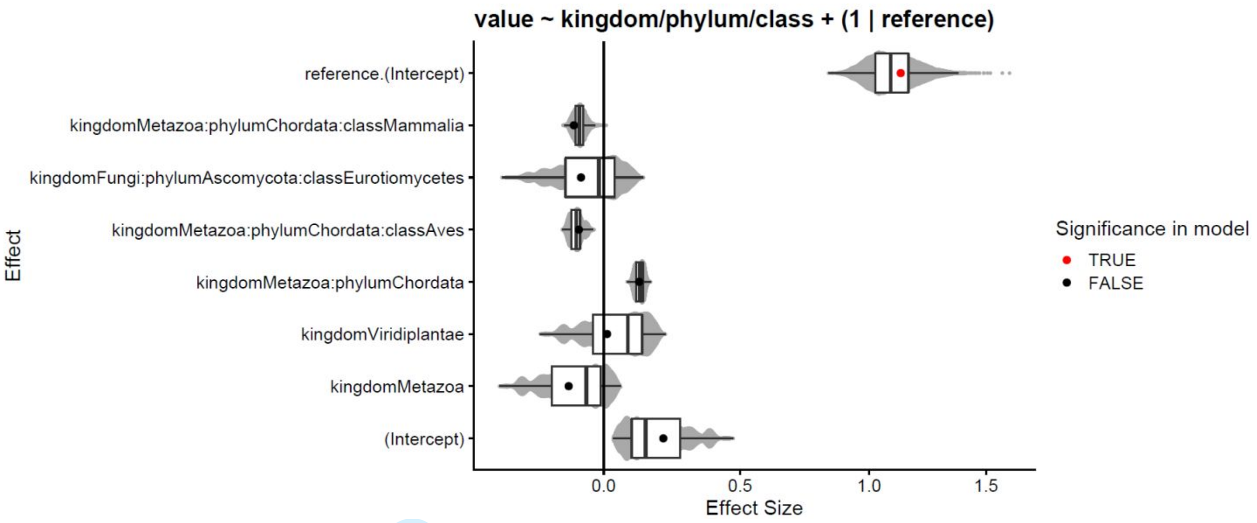


**Figure S11: Mixed model effect sizes for Model 7. Reducing the data to only include taxonomic classes with more than one study each, we find no significant differences between taxa in reported Patterson's *D*. Note, however, that effect size bootstraps don't overlap 0 for comparisons between Fish vs Mammals and Birds, Vertebrates vs Arthropods. Labels as in Figure S5.**

For Review Only

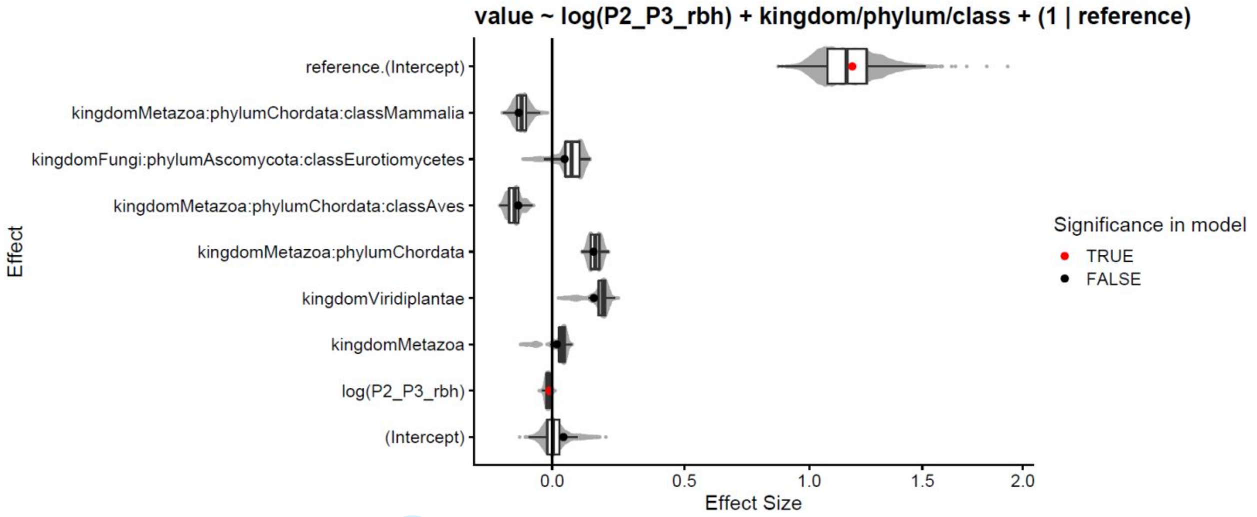


**Figure S12: Mixed model effect sizes for Model 8. Reducing the data to only include taxonomic classes with more than one study each, we find no significant differences between taxa in reported Patterson's *D*. Note, however, that effect size bootstraps don't overlap 0 for comparisons between Fish vs Mammals and Birds, Vertebrates vs Arthropods and Fungi vs Plants and Animals. Labels as in Figure S5.**

or Review Only

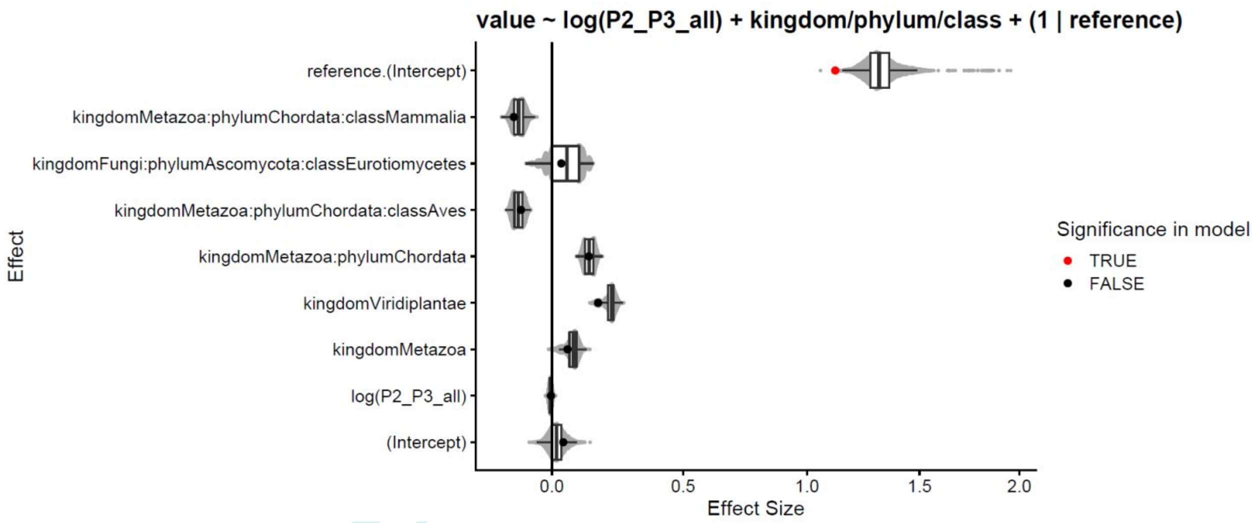


**Figure S13: Mixed model effect sizes for Model 9. Similar to Model 8, but using single genes for genetic distance. Labels as in Figure S5.**

For Review Only

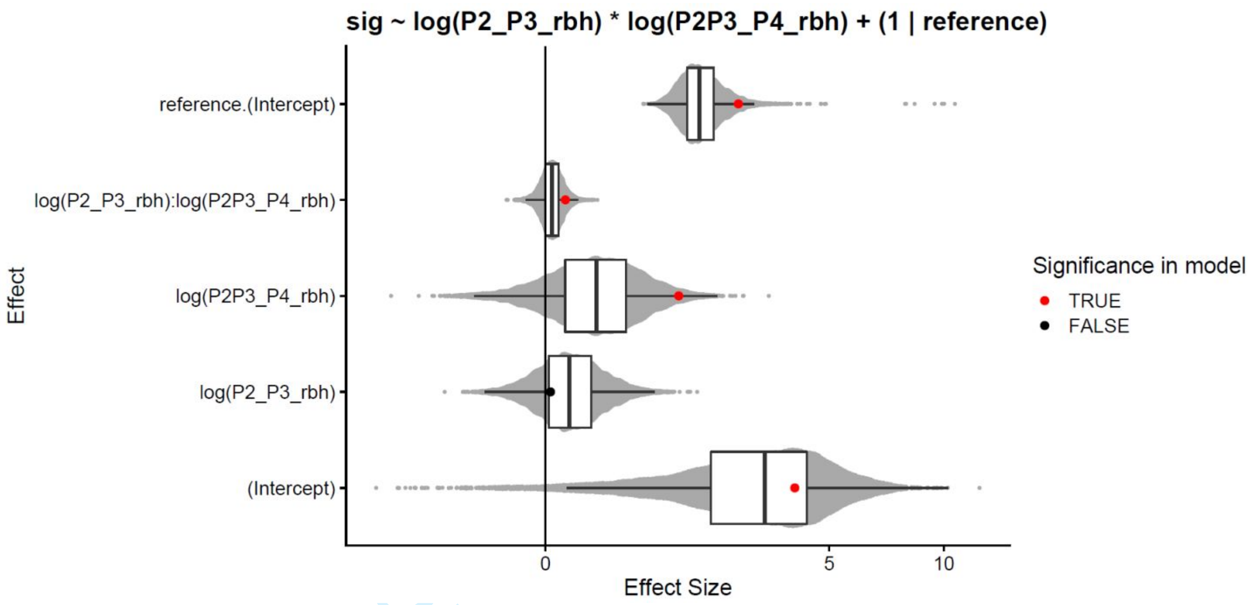


**Figure S14: Mixed model effect sizes for Model 10. Including distance to outgroup is significant, suggesting more distant outgroups result in a higher probability of detecting significant introgression. These effects are not significant after bootstrapping. However, note that our phylogenetic bootstrapping will exclude cases in which introgression between the same species pairs is reported using different outgroups, and so excludes some of the most informative cases.**

Review Only

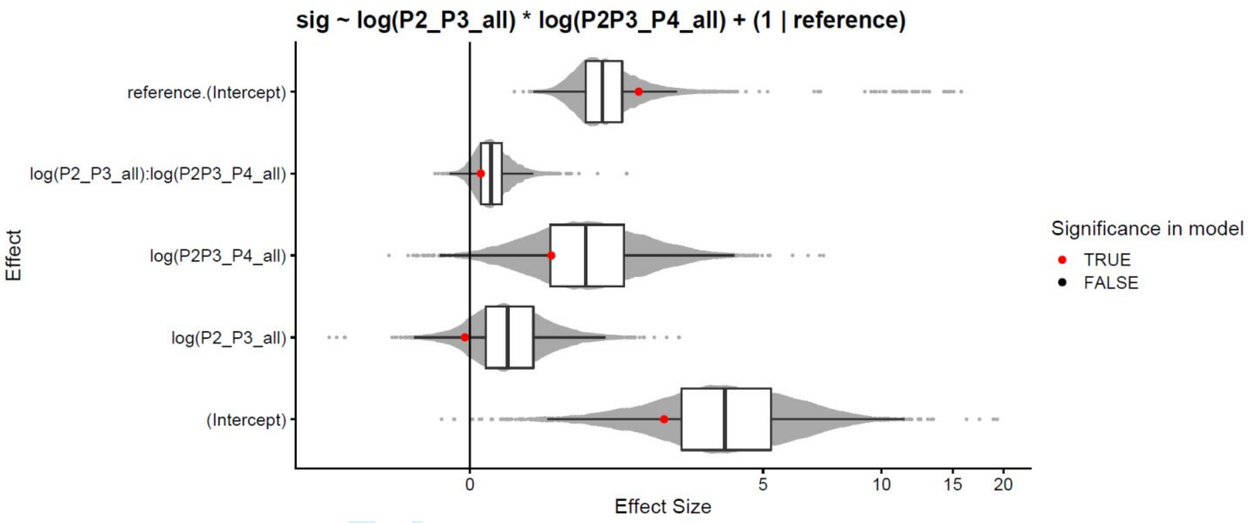


**Figure S15: Mixed model effect sizes for Model 11. As with Model 10, a different metric of genetic distance does not change the effect of distance to outgroup, but does change the direction of the effect of genetic distance between P2 and P3. As before, after bootstrapping these effects are not significant.**

For Review Only

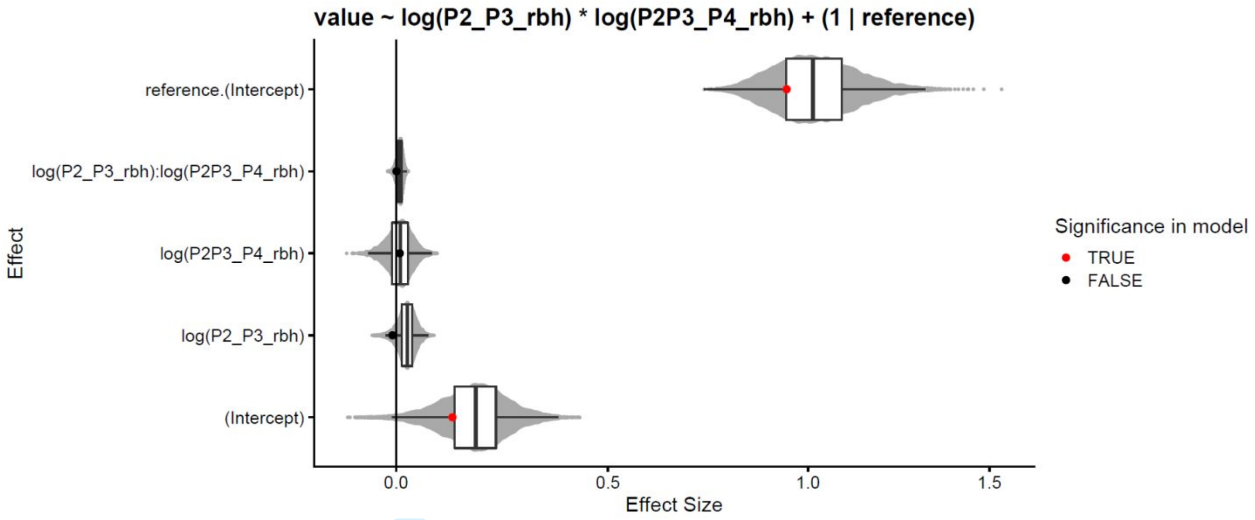


**Figure S16: Mixed model effect sizes for Model 12. Unlike significance of Patterson's *D*, there is no support for an effect of outgroup in the magnitude of Patterson's *D*.**

For Review Only

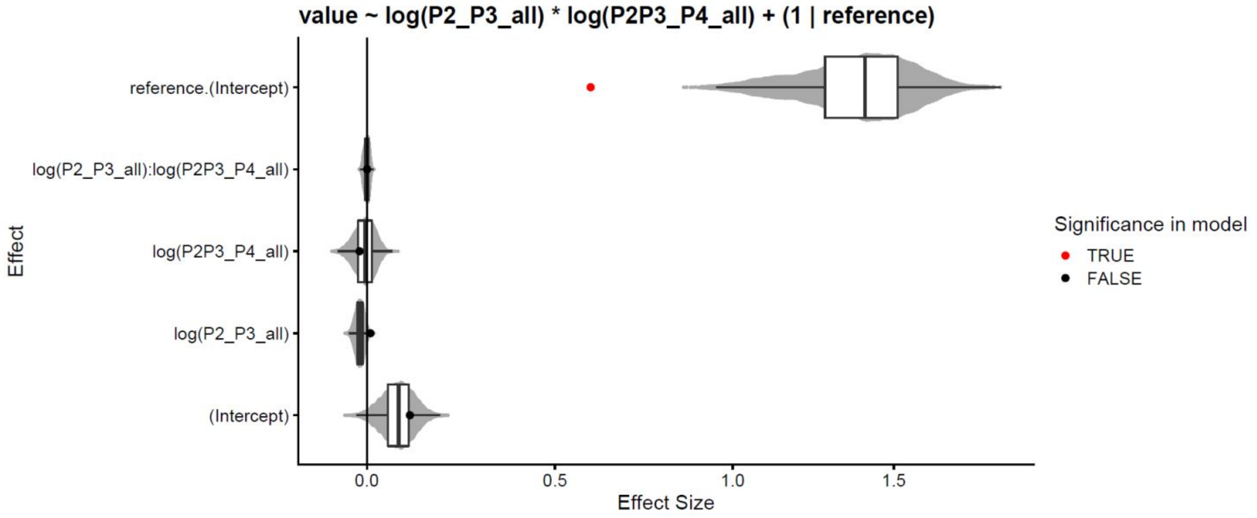


**Figure S17: Mixed model effect sizes for Model 13. As with model 13, using an alternate genetic distance measure does not support an effect of outgroup on the magnitude of Patterson's *D*.**

For Review Only

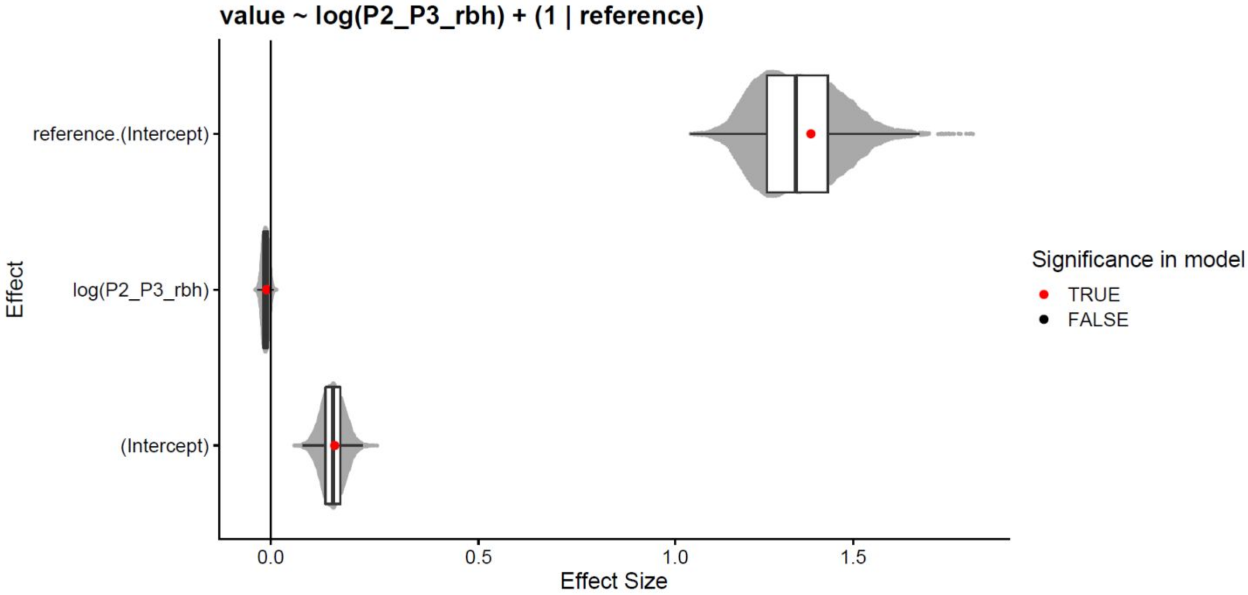


**Figure S18: Mixed model effect sizes for Model 14. Only retaining genetic distance, it is a significant predictor of the magnitude of Patterson's *D*, but bootstrap estimates overlap 0.**

Review Only

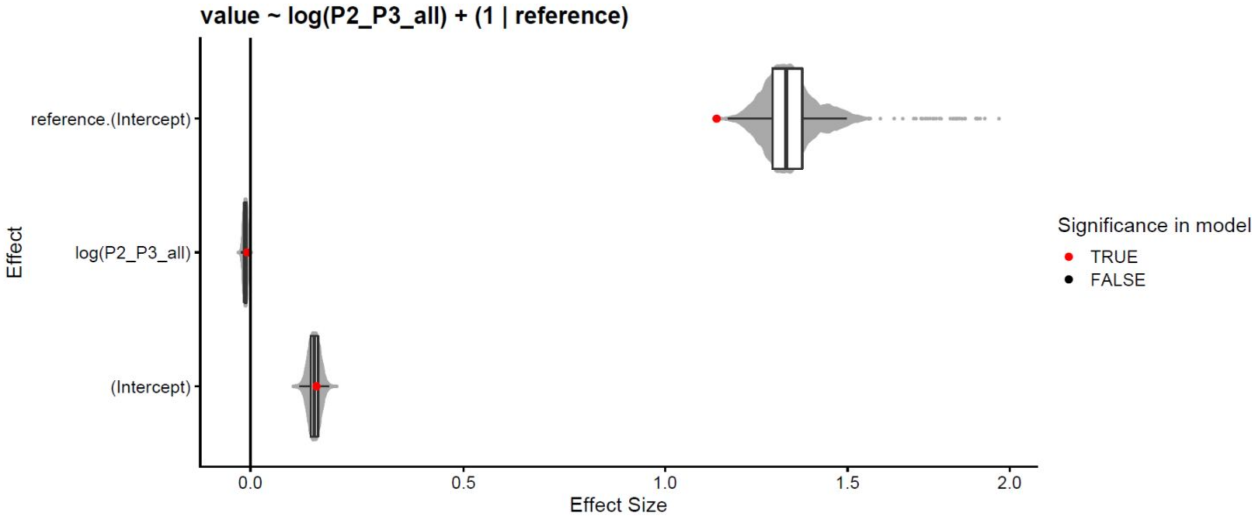


**Figure S19: Mixed model effect sizes for Model 15. Using single genes to calculate genetic distance, we see a similar pattern, although the bootstrap estimates are less variable.**

For Review Only

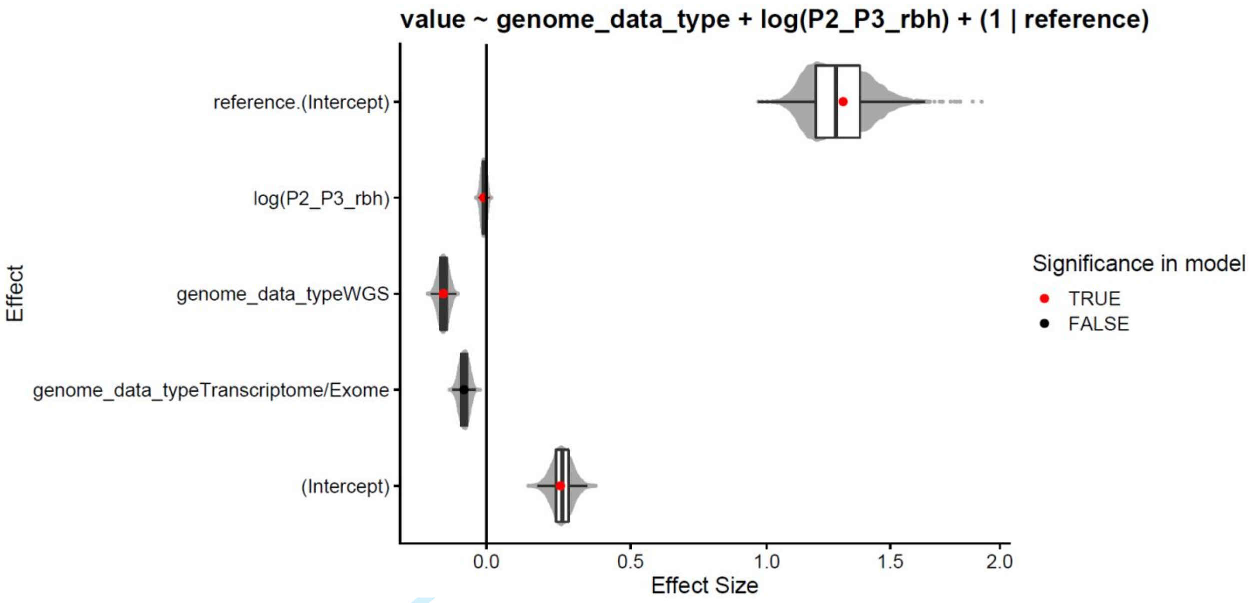


**Figure S20: Mixed model effect sizes for Model 16. Genomic data type is a significant predictor of the magnitude of Patterson's *D*. WGS is significantly different to RRS, while Transcriptome/Exome data is intermediate.**

Review Only

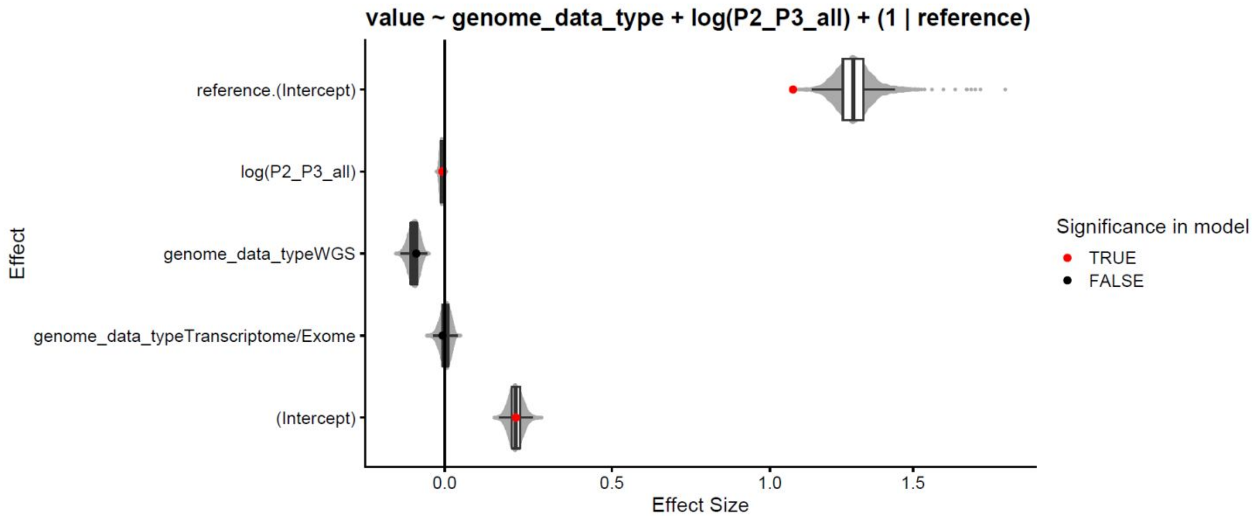


**Figure S21: Mixed model effect sizes for Model 17. Using single genes to calculate genetic distance, we see no significant effect of sequencing type, but note unequal reduction in data (only 5 RAD studies remain, for instance).**

For Review Only
